# Linking active rectal mucosa–attached microbiota to host immunity reveals its role in host–pathogenic STEC O157 interactions

**DOI:** 10.1093/ismejo/wrae127

**Published:** 2024-07-10

**Authors:** Zhe Pan, Yanhong Chen, Mi Zhou, Tim A McAllister, Tom N Mcneilly, Le Luo Guan

**Affiliations:** Department of Agricultural, Food and Nutritional Science, University of Alberta, Edmonton, AB T6G 2P5, Canada; Department of Agricultural, Food and Nutritional Science, University of Alberta, Edmonton, AB T6G 2P5, Canada; Department of Agricultural, Food and Nutritional Science, University of Alberta, Edmonton, AB T6G 2P5, Canada; Agriculture and Agri-Food Canada, Lethbridge Research Centre, Lethbridge, AB T1J 4B1, Canada; Moredun Research Institute, Penicuik EH26 0PZ, United Kingdom; Department of Agricultural, Food and Nutritional Science, University of Alberta, Edmonton, AB T6G 2P5, Canada; Faculty of Land and Food Systems, The University of British Columbia, Vancouver, BC V6T 1Z4, Canada

**Keywords:** mucosa-attached microbiota, STEC O157, community assembly, microbial co-occurrence network, host transcriptome

## Abstract

The rectal–anal junction (RAJ) is the major colonization site of Shiga toxin–producing *Escherichia coli* (STEC) O157 in beef cattle, leading to transmission of this foodborne pathogen from farms to food chains. To date, there is limited understanding regarding whether the mucosa-attached microbiome has a profound impact on host–STEC interactions. In this study, the active RAJ mucosa–attached microbiota and its potential role in host immunity–STEC commensal interactions were investigated using RAJ mucosal biopsies collected from calves orally challenged with two STEC O157 strains with or without functional stx2a (stx2a+ or stx2a−). The results revealed that shifts of microbial diversity, topology, and assembly patterns were subjected to stx2a production post-challenge and *Paeniclostridium* and *Gallibacterium* were the keystone taxa for both microbial interactions and assembly. Additional mucosal transcriptome profiling showed stx2a−dependent host immune responses (i.e. B- and T-cell signaling and antigen processing and presentation) post-challenge. Further integrated analysis revealed that mucosa–attached beneficial microbes (i.e. *Provotella, Faecalibacterium,* and *Dorea*) interacted with host immune genes pre-challenge to maintain host homeostasis; however, opportunistic pathogenic microbes (i.e. *Paeniclostridium*) could interact with host immune genes after the STEC O157 colonization and interactions were stx2a−dependent. Furthermore, predicted bacterial functions involved in pathogen (O157 and *Paeniclostridium*) colonization and metabolism were related to host immunity. These findings suggest that during pathogen colonization, host–microbe interactions could shift from beneficial to opportunistic pathogenic bacteria driven and be dependent on the production of particular virulence factors, highlighting the potential regulatory role of mucosa–attached microbiota in affecting pathogen–commensal host interactions in calves with STEC O157 infection.

## Introduction

Microbes colonize the gastrointestinal (GI) tract and interact with hosts for gut homeostasis maintenance, protection against pathogens, and immunity development [1, 2]. The epithelial surface of the GI tract is covered with mucus (a layer of a gel consisting of water, electrolytes, lipids, and proteins), which protects the epithelium barrier integrity and also plays a critical role by harboring mucosa–attached microbiota [3]. Different from fecal and luminal microbiota, region-specific mucosa–attached microbiota reside in the mucus adjacent to the host epithelium [3], where they contribute remarkably to gut barrier functions through roles in maturation of the host immune system [4], production of antimicrobial compounds for defense from enteric pathogenic bacteria colonization, and competition for nutrients [5, 6]. Although the majority of studies employed fecal/lumen samples to identify host–microbiota interactions under healthy/diseased conditions, several studies revealed that mucosa-attached microbiota interacted with hosts, regulating host–microbiome interactions and subsequently affecting host homeostasis [7–12]. For instance, microbial profiling of samples from rectal swabs of patients with colorectal cancer and healthy individuals revealed reduced relative abundance of *Bifidobacterium, Faecalibacterium*, and *Blautia* in mucosa–attached microbial communities in colorectal cancer patients, suggesting that the mucosa-attached microbiome has a role in host diseases [7].

Among enteric pathogen species, Shiga toxin–producing *Escherichia coli* (STEC) is a critical foodborne bacterium in humans, and STEC O157:H7 is the major serotype responsible for severe sequelae in humans, including hemolytic uremic syndrome and hemorrhagic colitis [13]. In particular, Shiga toxin 2 (Stx2), which is the main virulence factor in STEC O157:H7 [11] and is frequently associated with human illness, contains several variants, including stx2a, -b, -c, -d, -e, -f, and -g [14]. Cattle are the main reservoir for STEC O157, with the rectal–anal junction (RAJ) being the major colonization site. Cattle shedding more than 10^4^ colony-forming units (CFU) STEC per gram of feces are defined as “super shedders” (SS) and are the primary source of STEC transmission [15–19]. Commensal bacteria have been reported to inhibit STEC O157 colonization in the ruminant digestive tract through direct (i.e. competitive exclusion) and indirect (i.e. activation of host immune protection) mechanisms [20]. Recent studies have shown that varied microbial compositions and functions of mucosa-attached microbiota were affected by STEC O157 colonization in beef cattle [8]. For instance, RAJ mucosa–attached microbial profiles revealed that a total of 10 SS-specific microbes (e.g. *Acinetobacter*, *Corynebacterium*) with 12 altered functions relevant to amino acid and carbohydrate metabolism were identified in SS compared to uncolonized beef cattle. This finding suggests that upon STEC O157 colonization, mucosa–attached microbiota differed from healthy gut microbiota [8]. However, data are lacking with regard to how STEC O157, as a mucosa colonizer, interacts with RAJ mucosa–attached microbiota and how the mechanisms of such pathogen–microbiome interactions affect STEC O157 colonization.

Mucosal colonization of STEC O157 has been reported to cause host dysfunction in cattle [8, 21]. For instance, rectal transcriptomic profiles revealed reduced host innate and adaptive immune functions relevant to STEC O157 colonization in SS animals [21]. In the same cohort of beef cattle, correlations between differentially expressed host genes and predicted mucosa microbial functions (i.e. negative correlations between *S100A8* and microbial functions such as DNA replication proteins) were identified [8]. In addition, the expression of stx2 in STEC could restrict host gut epithelial regeneration and normal functions in calves [22]. Therefore, we speculated that pathogen colonization together with bacterial virulence factors in early life can alter the mucosa−attached microbiome with keystone microbes related to STEC O157 colonization that impact host immunity. The gut microbial community gradually becomes established after birth, and its assembly process is critical for shaping the structure and functions of the gut microbiome that influences host health [23, 24]. Therefore, in this study, we used STEC O157–challenged calves to assess if and how the active mucosa–attached microbiome affects host–pathogen interactions and to what extent the structures, assembly, and functions of the mucosa−attached microbiome shift in response to host–pathogen interactions.

## Methods

### Animal study and sample collection

All animal work was carried out at the Moredun Research Institute (MRI) under Home Office License 70/7914 granted by the UK Home Office under the Animal (Scientific Procedures) Act 1986 and was approved by the MRI animal care and ethics review committee.

Detailed information about the natural O157 strain (sourced from [25]), laboratory-reconstructed (RE) O157 strain, and calf experiments were described in previous research [22, 26]. The natural O157 strain contains both stx2a and stx2c prophages but only expresses stx2c (PT 21/28^*stx2a-stx2c*+^, Supplementary Fig. S1) due to the existence of insertion sequence ISEc8. However, the RE O157 strain contains and expresses both stx2a and stx2c prophages (RE 21/28*^stx2a + stx2c+^*, Supplementary Fig. S1). A total of 24 Holstein–Friesian veal calves entered the MRI at 3 weeks of age. Prior to the oral challenge, the fecal samples of calves were prescreened 5 times per week using immunomagnetic separation according to the manufacturer’s instructions (Dynabeads anti-STEC O157; 75 Invitrogen, Paisley, United Kingdom) to ensure negative STEC O157. The subsequent culture and quantitative reverse transcription PCR confirmed calves to be STEC O157, stx1, and stx2 negative before entering the trial (Supplementary Table S1). Calves were randomly assigned to three groups: phage type (PT) 21/28*^stx2a-stx2c+^*, wild type (WT; *n* = 6); RE 21/28*^stx2a + stx2c+^* (*n* = 7); and control (CT; *n* = 11). All calves were weaned and fed hay and calf starter through the trial and each challenge group of calves was allocated to different rooms at the MRI High Security Unit except the CT-group calves, which were conventionally on the MRI farm. Calves were orally challenged by orogastric intubation with ~10^9^ CFU of each STEC O157 strain in 10 ml of lysogeny broth. The RAJ tissue of each calf was biopsied at 3 days before challenge (T1), 7 days post-challenge (the highest fecal shedding level for challenge groups, T2), and 26 days post-challenge (decreased fecal shedding time, T5) of the trial. All tissues were stored at −80°C. The fecal sample collection and counting were performed (Supplementary Method 1).

### Mucosa-attached bacterial 16S rRNA gene amplicon sequencing and analysis

The RNA was extracted from tissue samples and cDNA was used to construct libraries for bacterial 16S rRNA gene amplicon sequencing (Supplementary Method 2). The raw sequence data were assigned to each sample according to the corresponding barcode and were processed using QIIME2 (version 2019.10) [27], and one sample from RE-T5 was removed due to the low-quality reads generated. Quality control, denoising, removal of chimeric sequences, and generation of amplicon sequencing variants (ASVs) were performed using the QIIME2 plugin DADA2 [28]. Taxonomic classification was performed in QIIME2 using a taxonomic classifier with the SILVA database (version 138) [27]. Only identified genera with a relative abundance >0.01% and a presence in at least half of the samples were included in further analysis. The Good’s coverage index was used to evaluate the adequacy of sequencing depth to generate bacterial profiles in each sample.

Alpha diversity was estimated using the Shannon index (evenness) and the number of ASVs (richness) as indices. Beta diversity was evaluated based on the Bray–Curtis distance to determine the similarities of active microbial profiles across times among the three groups. Principal component analysis (PCA) was adopted to identify the clustering patterns of the microbial profiles for the CT, WT, and RE groups from T1 to T5. Linear regression models were constructed to assess the relationship between alpha diversities and STEC O157 fecal shedding levels at T2 and T5 with Shannon/number of ASVs indices as independent variables and log10 fecal shedding levels as dependent variables (*P* value < .05 as significant). The PROC MIXED model in SAS (version 9.13; SAS Institute Inc., Cary, NC, United States) was used to analyze the effects of challenge and ages on the relative abundance of taxa at the phylum level.

### Microbial interactions within the active mucosa–attached microbiota using network analysis

Microbial networks were constructed using the relative abundance of microbial genera based on Spearman’s coefficient (absolute Spearman’s *R* > 0.6, *P* value < .05) and topological properties, including modularity (value >0.4, suggesting that the network has a modular structure) [29], average degree (the average number of connections per node) [30], and clustering coefficient (also termed transitivity, the degree to which nodes tend to cluster together) [31] were computed for each network. Then, two topological properties (average degree, clustering coefficient) representing node distributions were compared pairwise across time in microbial communities across the CT, WT, and RE groups using the Kolmogorov–Smirnov (KS) test in R [32].

Within-module connectivity (Zi) and among-module connectivity (Pi) were computed to characterize the topological role of nodes. Taxa that are highly connected with others both within and among modules (network hubs, Zi > 2.5; Pi > 0.62), within a module (module hubs, Zi > 2.5; Pi < 0.62), among different modules within a network (connectors, Zi < 2.5; Pi > 0.62), and less connected with other taxa (peripherals, Zi < 2.5; Pi < 0.62) [33] were classified.

Natural connectivity was used to measure the network stability based on the following algorithm:


$$ \mathrm{ave}\left(\mathrm{\lambda} \right)=\ln \left(\frac{1}{\mathrm{N}}\sum_{\mathrm{i}=1}^{\mathrm{N}}{\mathrm{e}}^{\lambda_{\mathrm{i}}}\right) $$

where $\mathrm{ave}\left(\mathrm{\lambda} \right)$ is the natural connectivity, N is the number of nodes in the network, and ${\mathrm{\lambda}}_i$ is the eigenvalue of the adjacency matrix. Up to 80% of the total nodes in each group were randomly removed from the adjacency matrix and ${\mathrm{\lambda}}_{\mathrm{I}}$ and $\mathrm{ave}\left(\mathrm{\lambda} \right)$ were re-calculated after each removal. The visualization of the natural connectivity was performed using the ggplot2 package in R.

### Assessment of mucosa-attached microbiota assembly patterns in response to STEC O157 challenge

Both deterministic factors (i.e. interspecies interactions, species traits, host) and stochastic factors (i.e. birth, death, colonization of microbes) can simultaneously occur and affect the assembly of microbial communities [34]. Determinism (deterministic factors) highlights strong selections imposed by environments and species interactions; however, stochasticity (stochastic factors) focuses on the random and unpredictable events affecting assembly [35]. The Raup–Crick distance (${\beta}_{RC}$) was used to assess the relative importance of stochastic/deterministic processes in the microbial assemblage. The ${\beta}_{RC}$ measures the extent to which the deterministic-driven assembly deviates from the assemblies based on null (stochastic) expectations: a value approaching −1 or 1 (${\beta}_{RC}$> 0.95 or ${\beta}_{RC}$ < −0.95) refers to the deterministic factors that drive microbial community assembly [36]. Whereas if ${\beta}_{RC}$ does not significantly deviate from 0 (−0.95 < ${\beta}_{RC}$ < 0.95), the community is considered a stochastic-driven assembly. A chi-square test was adopted to test the equality of proportions of processes belonging to either deterministic- or stochastic-driven assemblies (*P* value < .05 as a significant).

### Identification of microbial ecotypes in response to STEC O157 colonization and fecal shedding

The identification of three microbial ecotypes (generalist, specialist, neutralists) was based on niche breadth, which estimates the diversity of recourses used by an individual (or species) within a certain environment (Supplementary Method 3). The chi-square test was used to test the equality of numbers of specialized microbes across the CT, WT, and RE groups pre- and post-challenge (*P* value < .01 as significant). Microbial ecotypes among microbial assemblages that play a role in microbial interactions were identified (i.e. a taxon involved in both microbial networks and microbial assemblage). The relations between the relative abundance of identified dual-role microbes and both ${\beta}_{RC}$ and log_10_ STEC fecal shedding were assessed using linear regression models (*P* value < .05 as significanct).

### Identification of host immune-related pathways and interactions between host immune genes and mucosa-attached microbes

The host transcriptome profiling, bioinformatic analysis, and identification of altered pathways using gene set enrichment analysis (GSEA) are described in Supplementary Method 4. The PCA was used to identify clustering patterns of the host transcriptome for CT, WT, and RE from T1 to T5. Host immune pathways were then selected from the GSEA enrichment results, and only expressed genes among these selected pathways were considered for further analysis. Host genes from identified host immune-related pathways were correlated with the relative abundance of identified key microbes using the Spearman correlation (absolute *R* > 0.8 and *P* value < .01 as significant) among CT, WT, and RE at pre- and post-challenge.

### Predictions of microbial functionality and relations with host immunities

The microbial functionality for each treatment group from pre- to post-challenge was predicted using the “q2-picrust2” plugin in QIIME 2 [37]. Differential microbial pathways were identified based on MetaCyc [38] and compared between CT and WT, CT and RE, and WT and RE from pre- to post-challenge as well as between different time points for each treatment group using the Limma package in R with a false discovery rate–adjusted *P* value < .01 as significant. The differential microbial pathways were then correlated with genes involved in host immune-related pathways using Spearman correlations (absolute *R* > 0.8 and *P* value < .01 as significant).

## Results

### Assessment of fecal shedding level and potential calf growth effects on mucosa-attached microbiota and host transcriptome

Averages of 4.2 ± 1.10, 0.84 ± 1.01, 3.82 ± 1.42, and 2.68 ± 2.43 CFU/g log_10_ fecal STEC O157 were identified at WT-T2, WT-T5, RE-T2, and RE-T5, respectively, confirming that all orally challenged calves were successfully colonized following oral challenge. Unchallenged calves (CT) were negative for STEC O157 throughout the trial. By identifying clustering patterns of mucosa–attached microbiota at CT, the majority of samples from T1 clustered with samples from T2 and T5, with one exception of CT-T5 (Supplementary Fig. S2A). No clustering patterns were identified at CT from T1 to T5 for the mucosa–attached microbiota and host transcriptome (Supplementary Fig. S2A and B). The mucosa–attached microbial profiles at T1 were clustered and separated from T2 and T5 for both WT and RE (Supplementary Fig. S3). No clear separations were observed for the host transcriptome for both WT and RE from T1 to T5 (Supplementary Fig. S4).

### Similar structural variations of active rectal mucosa–attached microbiota in response to strain−specific STEC O157 colonization

A total of 11 375 ASVs were identified with an average of 160 ± 5 ASVs per sample from an average of 15 774 ± 3747 filtered paired-end reads (Supplementary Dataset 1). The Good’s coverage was >99.9% for all samples, indicating adequate sequencing depth for assessing microbial communities (Supplementary Dataset 2). A total of 13 phyla were identified across all groups, among which *Actinobacteria*, *Bacteroidota*, *Firmicutes*, and *Proteobacteria* were the most abundant (accumulated relative abundance accounting for >90% for each group, Supplementary Table S2). Among identified phyla, the relative abundances of *Bacteroidota* and *Actinobacteria* were affected by interactions between STEC challenge and calf age (*P_Bacteroidota_* = .02, *P_Actinobacteria_* = .04, Supplementary Table S2).

**Figure 1:**
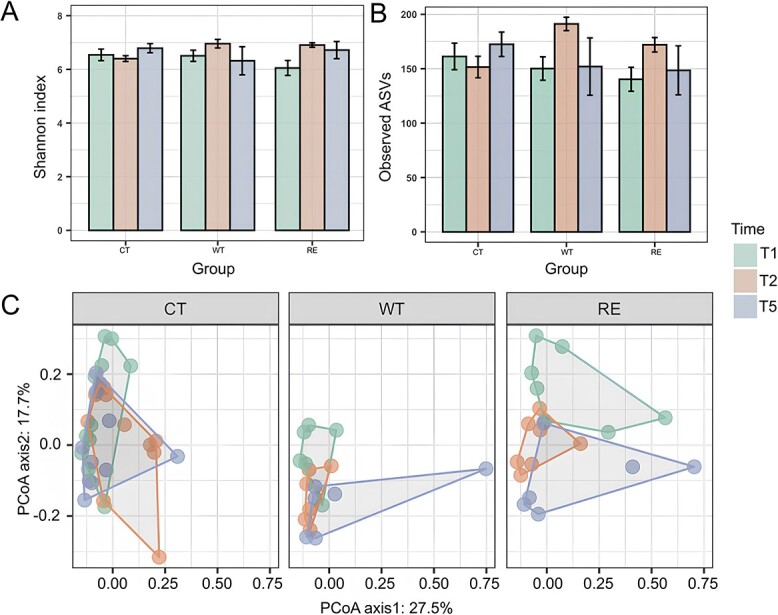
Comparison of diversity metrics for calves from control (CT), PT 21/28*^stx2a-stx2c+^* (WT) and RE 21/28*^stx2a + stx2c+^*(repairment, RE) from T1 to T5. The Shannon index (A) and observed ASVs (B) were used to estimate the evenness and richness across three groups with three different colors of bars representing samples collected from T1, T2, and T5. The horizontal bars within boxes represent medians. The KS test was used to assess if the shifts of alpha diversities from pre- to post-challenge were significantly different (*P* value ≤ .01 as a significance) or shared a similar trend of changing patterns (.01 < *P* value < .1). The Kruskal–Wallis test was used to determine whether indices between any two groups at one certain time point were significantly different (*P* value ≤ .05). Principal coordinate analysis (PCoA) was used for the visualization of the Bray–Curtis distance. The ANOSIM (analysis of similarities) was used to test for the similarity of clustering patterns among different ages within each group. Differences were considered significant at *P* ≤ 0.05.

Both the Shannon index and the number of ASVs in the WT and RE calves exhibited similar change patterns: increase from T1 to T2 and then decrease from T2 to T5 (All KS test *P* values > 01, Fig. 1A and B). However, the shift in alpha diversity in the CT differed from those in the WT and RE calves, with both indices being the lowest at CT-T2 (Fig. 1A and B). Furthermore, all three groups differed (*P*_Shannon_ < .01, *P*_Number of ASVs_ = .03) in the Shannon and the number of ASVs indices at T2. Similarly, the Bray–Curtis distance of microbial similarity showed a separation of rectal bacteria between T1 and T5, but not between T2 with T1 or T5 in both the WT and RE groups. However, this pattern was not apparent in the CT group (Fig. 1C). The ANOSIM analysis identified the significant interaction effect between calf age and STEC challenge on microbial similarities (*P*_Age*Challenge_ = .01, *P*_Age_ = .21, *P*_Challenge_ = .36). In addition, relationships between alpha diversities and log_10_ fecal shedding levels varied in WT and RE (Fig. S5). In particular, both the Shannon and number of ASVs indices were negatively correlated to log_10_ fecal shedding at WT-T2 (*P*_Shannon_ = .04, *P*_Number of ASVs_ = .02, Fig. S5A), however, no correlation was observed with the RE.

### Differential microbial interactions in active mucosa-attached microbiota in response to strain-specific STEC O157 colonization

**Figure 2:**
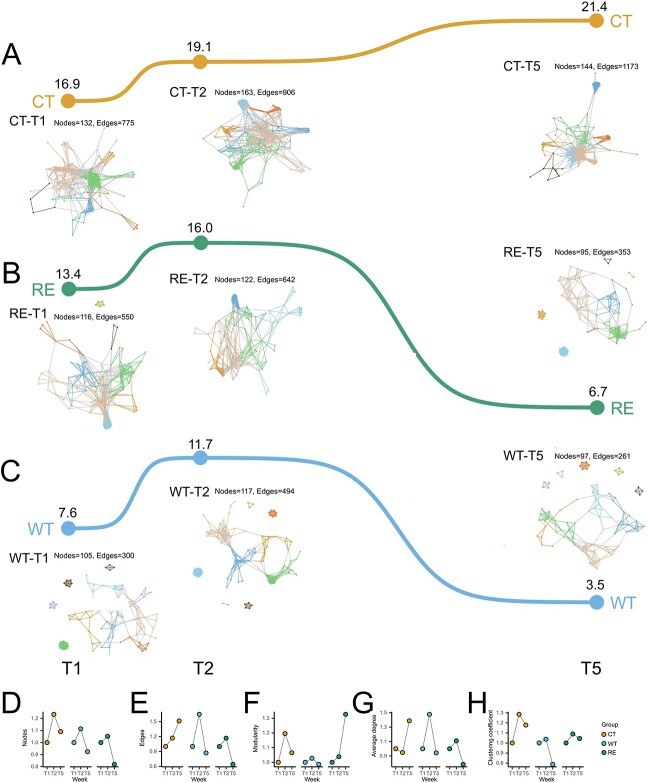
Dynamic microbial interactions were revealed by microbial co-occurrence network analysis in CT (A), RE (B), and WT (C) from T1 to T5. The line on top of co-occurrence networks represents network stabilities with values near the line. The log fold change relative to the value at T1 of each network property including nodes (D), edges (E), modularity (F), average degree (G), and clustering coefficient (H) were visualized for each group.

The patterns of microbial co-occurrence networks and properties were different among the three groups. The network stability was continuously increased from T1 to T5 in CT (Fig. 2A), while it was highest at T2 and lowest at T5 for WT and RE (Fig. 2B, C). The number of nodes (microbial taxa) had similar changing patterns in all three groups during the experimental period, which increased from T1 to T2 and decreased from T2 to T5 (Fig. 2D, Supplementary Table S3). The number of edges peaked at T2 for both WT and RE, while it increased from T1 to T5 in CT (Fig. 2E). The modularity (the index measure of the strength of division of a network into modules, with a value >0.4 suggesting the network has a modular structure [29]) was greater than 0.4 for CT, WT, and RE during the experimental period (Fig. 2F). The average degree (an index that measures the number of edges connected to a node) was the highest at T2 for both WT and RE, while it was the highest at T5 for CT (Fig. 2G). For clustering coefficients, the highest value was observed at T2 for all three groups (Fig. 2H), while KS tests showed differences (*P* value < .01) of average degrees and clustering coefficients among three groups from T1 to T5.

From the network modularity perspective, network hubs and module hubs were not identified in CT, WT, and RE. An average of 2% (ranging from 0 to 3.8%) of total nodes were designated as connectors; however, an average of 98% ± 0.4% of total nodes were classified as peripherals for the CT, WT, and RE groups (Supplementary Table S4).

### Active mucosa–attached microbiota assembly patterns shifted during the STEC O157 colonization

**Figure 3:**
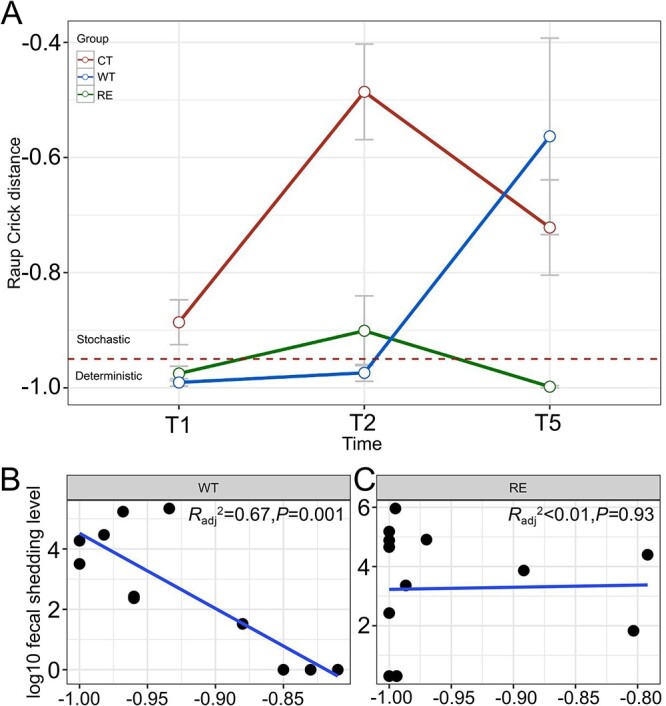
Microbial assembly patterns determined by Raup–Crick distance and its relations with log _10_ STEC O157 fecal shedding level. The dynamics of microbial community assembly patterns in three groups from T1 to T5 (A). The dotted horizontal line represents the boundary line (= −0.95) that separates assembly patterns being more stochastic−driven (above) or deterministic−driven (below). The linear regression model shows the relations between Raup–Crick distance and log_10_ STEC O157 fecal shedding in the WT (B) and RE (C) groups.

The Raup*–*Crick (${\beta}_{RC}$) distance revealed that microbial community assembly patterns in CT were consistently stochastic driven (Fig. 3A) as confirmed by the chi-square test (Supplementary Table S5), while it transitioned from a deterministic driven (T1 and T2) to a stochastic driven (T5) assembly in WT (Fig. 3A, Supplementary Table S5). For RE, the microbial community assembly was stochastic driven at T2 (Fig. 3A), while a deterministic process made the major contribution (86% to 100%) to assemblies at T1 and T5 (Supplementary Table S5). In addition, ${\beta}_{RC}$ was negatively correlated with log_10_ STEC O157 fecal shedding in WT as ${\beta}_{RC}$ was increased to become more stochastic driven with lower O157 fecal shedding (*R*^2^_adj_ = 0.67, *P* value < .01, Fig. 3B), while ${\beta}_{RC}$ measured for RE was not related to log_10_ STEC O157 fecal shedding (Fig. 3C).

### Specific mucosa–attached microbes identified as key bacteria taxa in both microbial interactions and assembly during the STEC O157 colonization

**Figure 4:**
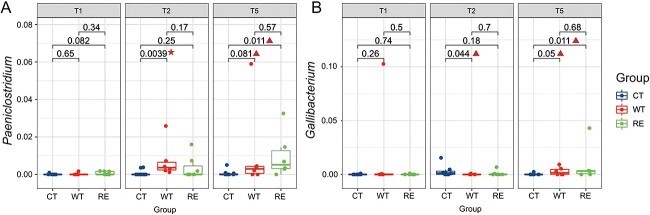
The comparison of the relative abundance of *Paeniclostridium* (A) and *Gallibacterium* (B) across each group from T1 to T5. *P* value < .01 as significance (marked as a triangle) and *P* value < .05 as a trend being significant (marked as a star).

A range of 6 to 29 (6%–18%) generalists and 5 to 19 (5%–14%) specialists were identified for all three groups from T1 to T5 (All *P* values >.10, Supplementary Table S6 and Supplementary Fig. S6). Two bacterial taxa (*Paeniclostridium* and *Gallibacterium*) were the only microbial ecotype (specialists) designated as network connectors. Particularly, the relative abundance of both *Paeniclostridium* and *Gallibacterium* were similar among CT, WT, and RE pre-challenged calves (Fig. 4A and B), but it was higher in WT compared to CT and RE calves at T2 (*P* value < .01, Fig. 4A). At T5, the relative abundance of *Paeniclostridium* showed a tendency of being higher in both WT and RE compared to CT (*P*_WT vs CT_ = .08, *P*_RE vs CT_ = .01, Fig. 4A). At T2, the relative abundance of *Gallibacterium* showed an increasing trend in CT compared to WT; however, it did not differ between CT and RE (Fig. 4B). At T5, the relative abundance of *Gallibacterium* showed an increasing trend in both WT and RE compared to CT at T5 (*P*_WT vs CT_ = .05, *P*_RE vs CT_ = .01, Fig. 4B). The relative abundance of *Paeniclostridium* was positively correlated with ${\beta}_{RC}$ (*R*^2^_adj_ = 0.68, *P* = .03) at T2 and negatively correlated with ${\beta}_{RC}$ at T5 (*R*^2^_adj_ = 0.66, *P* = .05) at WT (Fig. S7A top left and top right). For the relative abundance of *Gallibacterium,* only samples at T2 tended to be positively correlated with ${\beta}_{RC}$ (*R*^2^_adj_ = 0.36, *P* = .09, Supplementary Fig. S7B bottom left).

### Mucosa−attached beneficial and pathogenic microbes harbor varied interactions with host immune genes from pre- to post-challenge

The GSEA analysis of rectal transcriptomes revealed altered pathways related to host immunities (defined as host immune−related pathways, Supplementary Table S7). Three host immune−related pathways, including the MAPK signaling pathway, antigen processing and presentation, and the T-cell receptor signaling pathway, were upregulated at T2 compared to both T1 and T5 in WT, with the B-cell receptor signaling pathway being the only pathway upregulated at T2 compared to T1 in WT (Supplementary Fig. S8A). For RE, both T-cell and B-cell receptor signaling pathways were upregulated at T5 and T2 compared to T1. Four pathways, including antigen processing and presentation, the chemokine signaling pathway, intestinal immune network for IgA production, and the natural killer cell–mediated cytotoxicity were upregulated at T5 compared to T1 (Supplementary Fig. S8B).

**Figure 5:**
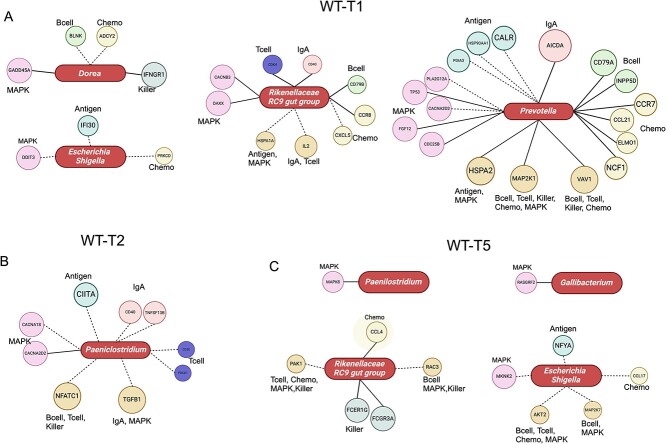
Interactions between selected microbes and host immune gene expressions in WT at T1 (A), T2 (B), and T5 (C). The solid line and dotted line refer to positive and negative interactions, respectively. The rod shape represents mucosa−attached microbes, and the circle refers to host immune−related genes with divergent colors representing different host immune−related pathways. For certain genes that were involved in more than one pathway, all pathways were labeled. Antigen, Bcell, Chemo, MAPK, Killer, IgA, Tcell refer to antigen processing and presentation, B-cell receptor signaling pathway, chemokine signaling pathway, MAPK signaling pathway, natural killer cell mediated cytotoxicity, intestinal immune network for IgA production, T-cell receptor signaling pathway, respectively.

**Figure 6:**
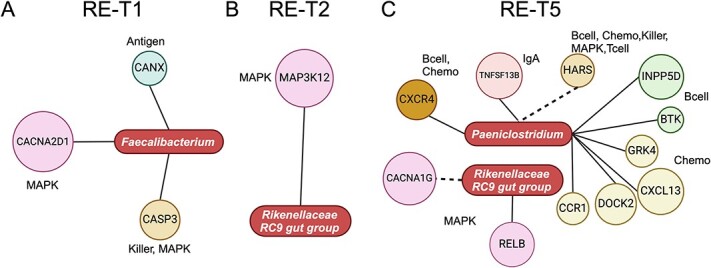
Interactions between selected microbes and host immune gene expressions in RE at T1 (A), T2 (B), and T5 (C). The solid line and dotted line refer to positive and negative interactions, respectively. The rod shape represents mucosa−attached microbes, and the circle refers to host immune–related genes with divergent colors representing different host immune−related pathways. For certain genes that were involved in more than one pathway, all pathways were labeled. Antigen, Bcell, Chemo, MAPK, Killer, IgA, and Tcell refer to antigen processing and presentation, B-cell receptor signaling pathway, chemokine signaling pathway, MAPK signaling pathway, natural killer cell–mediated cytotoxicity, intestinal immune network for IgA production, and T-cell receptor signaling pathway, respectively.

We further assessed interactions between active mucosal attached microbes and expressions of genes involved in host immune−related pathways (Supplementary Figs. S9 to S11). At WT-T1, the relative abundances of *Prevotella,* the *Rikenellaceae RC9 gut group,* and *Dorea* were significantly associated with host immune gene expressions (Fig. 5A). In particular, the relative abundance of *Prevotella* (mean ± SD, 0.2 ± 0.001) was mostly associated with host immune gene expressions (Fig. 5A). Only negative correlations were identified between the relative abundance of *Paeniclostridium* (0.7 ± 0.008) and host gene expressions at WT-T2 (Fig. 5B). At WT-T5, the relative abundance of the *Rikenellaceae RC9 gut group* (0.7 ± 0.003) and *Escherichia–Shigella* (7.8 ± 0.17) were mostly associated with host immune gene expressions (Fig. 5C). For RE, only the relative abundance of *Faecelibacterium* (4.7 ± 0.04) and the *Rikenellaceae RC9 gut group* (1.7 ± 0.009) was positively correlated with host gene expressions at T1 and T2, respectively (Fig. 6A and B). At RE-T5, the relative abundance of *Paeniclostridium* (1.0 ± 0.01) was mostly associated with host immune gene expressions (Fig. 6C).

### Predicted bacterial functions related to altered host immune–related pathways in response to *E. coli* O157 challenge

**Figure 7:**
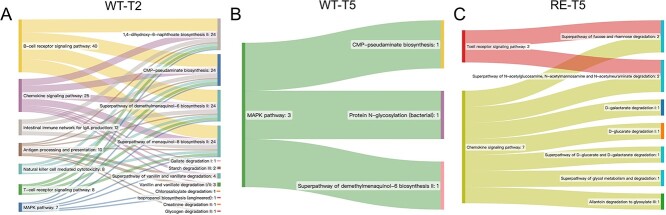
Sankey diagram showing the significant linkage between host immune−related pathways and predicted microbial functions for WT-T2 (A), WT-T5 (B), and RE-T5 (C). The value adjacent to each host pathway/ microbial function represents genes/microbes involved in this pathway/microbial functions relating to microbial functions/host pathways, respectively.

A number of 15, 15, 3, and 11 predicted microbial pathways were identified as DE pathways in CT-T2, WT-T2, WT-T5, and RE-T5, and no DE pathways were enriched for other time points (Supplementary Fig. S12 and Supplementary Table S8). There was no overlap for predicted pathways from CT-T2 and WT-T2 as well as WT-T5 and RE-T5 (Supplementary Fig. S12 and Supplementary Table S8). Three DE pathways, including CMP-pseudaminate biosynthesis, protein *N*-glycosylation (bacterial), and superpathway of demethylmenaquinol-6 biosynthesis II were overlapped for both WT-T2 and WT-T5 (Fig. 12B and C). Further analysis revealed that host genes involved in the B-cell receptor signaling pathway followed by the chemokine signaling pathway were mostly associated with bacterial functions at WT-T2 (Fig. 7A); however, only the MAPK signaling pathway was associated with bacterial functions at WT-T5 (Fig. 7B). For RE-T5, the chemokine signaling pathway followed by the T-cell receptor signaling pathway was related to bacterial functions (Fig. 7C).

## Discussion

Our study assessed an active hindgut mucosa–attached microbiota, its compositional, interactive, and assembly shifts, and how such dynamic shifts interacted with host immune gene expressions in response to strain-specific STEC O157 colonization. We identified the keystone taxa involved in both microbial interactions and assembly during the colonization with stx2a+ and stx2a− STEC O157 strains with possible mechanisms of mucosa-attached microbes regulating host–pathogen interactions.

In previous studies mucosa samples were collected based on the gut region of interest and used varied approaches to ensure the proper collection of mucosa samples, including direct collection using rectal swabs to study rectal mucosa–attached microbiota [7, 8], biopsies obtained endoscopically to study colon mucosa [9], upper GI endoscopy [10], collection of mucosa from the whole GI tract using a slide in mice [11], and epithelium scrapings from the whole GI tract in pigs [12]. In our study, a mildly invasive biopsy tool was adopted to collect RAJ tissue from each calf to ensure that microbiome studies were sourced from the mucosa instead of the lumen. As opposed to previous studies which used DNA-based amplicon sequencing to investigate mucosa-attached microbiome interactions [8, 39], the current study used an RNA-based approach to investigate the rectal mucosa–attached microbiota interactions occurring as a result of the STEC O157 challenge. The mucosa-attached microbial amplicon sequencing in beef cattle revealed that 5-7N15 (6.9% ± 2.4%, belonging to the family of *Bacteroidaceae*), *Prevotella* (4.7% ± 4.1%), and *Ruminococcus* (3.8% ± 6.1%) were the most abundant classified genera [8]. Another DNA-based amplicon sequencing suggested that *Treponema* was the most abundant classified genera (9.1%) in the rectal mucosa of dairy cattle [39]. However, *UCG.005* (30%) and *UCG.010* (15%) from both the *Oscillospirales* order and *Christensenellaceae R7 group* (9%) were the most abundant genera in all calves in our study. However, whether fecal microbiota at the RNA level share similar compositions with mucosa microbiota using the same cohort of calves in our study is unclear. Since the objects and sequencing of each study are inherently different (i.e. using British × Continental feedlot cattle and sequencing of the V1-V3 region [8] or using Holstein dairy cattle aged 5 years and sequencing of the V3-V4 region [39]), it is difficult to make direct comparisons among studies. However, as RNA-based amplicon sequencing is capable of revealing transcribed microbial communities, further microbiome studies using RNA-based amplicon sequencing for both fecal and mucosa microbiomes should be encouraged to identify the contribution of “active” members of these microbial consortia.

Our study revealed similar microbial communities in CT calves from T1 to T5, suggesting that calf development played a limited role in affecting mucosa−attached microbial profiles. Additionally, nonclustering host transctiptomic patterns from T1 to T5 as well as no differential host pathways identified at CT further suggested that calf development during our trial played a limited role in affecting the host RAJ mucosal transctiptome. It is possible that our observation period (~29 days) was too short to observe how calf growth development affected host−microbiome interactions. Although calves were negative for STEC O157 when entering the trial, the differential mucosa−attached microbial stabilities at T1 among CT, WT, and RE led to the inference that the initial microbial communities may have differed before challenge. Therefore, future studies targeted at identifying the impact of STEC O157 on gut commensals should try to reduce variation in the host intestinal microbiome.

Our study identified potential mechanisms of active mucosa–attached microbiota in regulating STEC O157 colonization. The profiles and diversities of mucosa–attached microbiota were altered after STEC O157 colonization. Similar to the findings of other SS studies [40, 41], we found higher alpha diversity in calves with higher fecal shedding levels. This observation likely reflects the ability of STEC O157 to be a successful competitor not only within gut lumen bacterial communities [42], but also in the mucosa–attached microbiota. At T2, STEC O157 may deprive commensal bacteria of nutrients, enabling STEC O157 to reach peak population levels. As a result, rectal mucosal attached microbial communities may become more diverse to regain the space and resources to maintain their homeostasis and to repel pathogens. This process may be reflected by the increase in alpha diversity at peak shedding in both groups challenged with STEC O157. Once STEC O157 colonization is limited, the microbiota shifts back to a less diverse community as reflected by the reduced alpha diversity at T5. Similarly, this same pattern was observed for the beta diversity of bacterial communities in calves challenged with STEC O157 compared with calves that were not. This observation suggests that active mucosal microbial communities could respond to rectal mucosal STEC O157 colonization, highlighting the importance of examining active mucosal microbiota for pathogen colonization in beef cattle. In addition, we observed that microbial interactions within mucosa–attached microbes and community stabilities were shifted after O157 colonization. It is possible that during O157 colonization, the production of certain microbial metabolites served as regulators between STEC O157 and mucosa−attached microbes with prominent bacterial taxa increasing community stabilities. For instance, *Prevotellaceae UCG.003*, a member of the Bacteroidota*,* was more abundant in the PT 21/28*^stx2a-stx2c+^* group (0.003 ± 0.003) than the RE 21/28*^stx2a + stx2c+^* group (0.001 ± 0.001, *P* value < .05) at T2. Members of the Bacteroidota are known to produce butyrate, which can inhibit the growth and colonization of *E. coli* and is critical to microbial interactions [43, 44]. Hence the higher abundance of *Prevotellaceae* at WT-T2 may confirm the inference that the higher level of butyrate produced in the gut against natural STEC O157, which is incapable of producing functional stx2a and augmenting microbial community stabilities. A previous study revealed that stx2a production in STEC could restrict epithelial regeneration and enhance rectal antibody responses (i.e. H7, Tir, EspA, and Intimin-specific IgA), suggesting that stx2a production may compromise the host immune system and affect host homeostasis [22]. Another study revealed that stx2 expression in STEC affected rectal fecal microbial communities by altering interactions and keystone taxa, indicating close associations between stx2 and gut commensals [19]. However, whether mucosa–attached microbial metabolites could play an intermediate role between stx2a and host immunity alternations is unclear. Metabolomic analysis targeted at assessing microbial metabolite shifts during this process could help improve our understanding of the nature of STEC O157–gut commensal interactions.

The microbial community assembly is vital for the successful establishment and maintenance of microbial populations and responds to environmental changes and host factors [45–47]. For instance, a recent study of the rumen of adult dairy cows revealed that stochasticity could shape long-term rumen microbiome development in cattle [47]. However, there is a lack of such research in the mucosa−attached microbiome and in bovines during the pathogen colonization. We found that patterns and dynamics of rectal mucosa microbial community assembly could be affected by STEC O157 colonization together with stx2 subtype differences. In particular, the production of stx2a but not stx2c shifted assembly patterns from deterministic driven to stochastic driven at T2. During the community assembly process, microbes are affected by both stochastic and deterministic factors and can interact with each other, leading to the specialization of certain microbial taxa within the niche [45, 48]. As a result, the production of stx2a in RE may have minimized the effects of microbial interactions and responses to gut environmental changes. The microbial community assembly was driven by the stochastic process in unchallenged calves, suggesting that age, which was previously reported to be a deterministic factor affecting microbiome assembly [47], plays a limited role in affecting hindgut mucosa microbiome assembly. We found that assembly patterns differed in challenge groups compared to the control group at T1 when all calves were weaned and fed under the same environmental conditions, indicating that individualized responses play a critical role in gut microbiome assembly.

In our study, key bacterial taxa were identified to be related to STEC O157 colonization and rectal mucosa–attached microbial interactions as well as assembly. The *Paeclostridium* and *Gallibacterium* were network connectors dominating microbial interactions and simultaneously being assembly specialists for both WT and RE. Assembly specialists harbor narrow ecological niches but are more affected by deterministic processes due to their preferences and sensitivities to external environmental conditions [49, 50]. Our results confirmed that the relative abundance of opportunistic pathogenic *Paeniclostridium* and *Gallibacterium* increased after STEC O157 colonization. And such variations can be STEC strain dependent, as reflected by their increased relative abundance increased at T2 in WT but at T5 in RE. Correspondingly, the expression of Semaphorin-6A (*SEMA6A*), the gene encoding for the receptor of exotoxin TcsL in *Paeniclostridium* [51]*,* was higher in rectal tissue of WT compared to unchallenged calves at T2, while this increase was not observed for RE calves at T2 (Supplementary Fig. S13). Our study revealed the expression of *SEMA6A* as the receptor of *Paeniclostridium* that was affected by the production of stx2a and STEC O157 colonization. Further evidence is that the *Paeclostridium* exhibited varied interactions with community assembly patterns in WT post-challenge, while no effects were found in RE pre- and post-challenge. As a bacterial pathogen, *Paeclostridium* can produce hemorrhagic toxins causing acute infectious disease in humans and animals [52, 53]. The increased relative abundance of this bacterial taxon after STEC O157 colonization in WT calves may suggest a potential mutualism between *Paeclostridium* and STEC, and such mutualism could augment the survival and proliferation of these two bacteria. However, the expression of stx2a did not significantly affect interactions between the relative abundance of *Paeclostridium* and microbial assembly at RE-T2, suggesting that stx2a expression may be a negative signal that inhibits interactions for other microbes, causing the absence of interactions observed for RE at T2. *Gallibacterium*,the other network connector that is designated to assembly specialists, is an opportunistic pathogen in animals [54]. However, no significant relationships with community assembly were found for *Gallibacterium*, suggesting that this microbial taxon could play a trivial role in affecting RAJ mucosal microbial assembly.

Pathogen-driven host responses were identified, indicating that the stx2 subtype in STEC could affect the host transcriptome and thereafter strain-specific host immune responses. This finding is similar to those in previous studies reporting enhanced cellular and humoral immune responses in calves challenged with stx2-positive STEC O157 [55]. In addition to pathogen−driven host responses, the mucosa–attached microbiota is also a key component in regulating host immune functions [8]. For instance, an on-farm survey of collected mucosa samples from RAJ of beef cattle naturally colonized with STEC revealed that expressions of host immune genes (i.e. *S100A* gene families) were positively associated with the relative abundance of mucosa-attached *Pseudomonas, Clostridium, Blautia,* and *Dorea*, with predicted microbial pathways relating to the replication of microbial genetic materials and metabolism [8], suggesting the critical role of mucosa−attached microbes in regulating host–pathogen interactions. Among the gut microbiota, commensal organisms such as *Prevotella* [56, 57]*,* the *Rikenellaceae RC9 gut group* [58, 59]*, Fecalibacterium* [60], and *Dorea* [61] were reported to be beneficial to the host. For instance, *Dorea* has been reported to be one of the most abundant genera in nonshedders [62] and is a beneficial butyrate-producing bacteria in the gut that enhances tight junctions of the epithelium [63]. Indeed, we found that interactions between beneficial mucosa−attached microbes and host immunities were intensive before pathogen colonization in both WT and RE. For example, *Prevotella,* a mucosa−attached commensal organism, is positively associated with high fiber consumption and can distinctively modulate host immune responses and gut barrier functions in human epithelial cells [64]. Our findings that host immune−related pathways showed intense positive interactions with *Prevotella* pre-challenge suggest that beneficial microbes are positively related to host immunities when the host remains in homeostasis. However, STEC O157 colonization shifted such beneficial microbe–host immune gene interactions for both WT and RE. The aforementioned *Paeniclostridium*, the identified microbes involved in both microbial interactions and assembly, showed significant negative interactions with host immune genes at T2 in calves challenged with PT 21/28*^stx2a-stx2c+^* challenged calves, suggesting that *Paeniclostridium* is a critical microbe involved in host–pathogen interactions.

The functional analysis further revealed that predicted bacterial functions associated with host immune responses and their changes during the STEC infection were stx2 subtype dependent. A larger number of predicted microbial pathways relevant to STEC colonization were identified at WT-T2 and RE-T5, a finding that also corresponds to changes in host immune responses. In particular, enhanced microbial functions may be related to observed increased microbial modularity at RE-T5, which refers to topological structures of microbial taxa that tend to be modular for similar microbial functions [19]. The CMP-pseudaminate is a sialic acid–like sugar that is unique to microorganisms as the constituent of cell surface glycoconjugates (i.e. lipopolysaccharide) and can influence bacterial pathogenesis through immune evasion [65, 66]. The upregulated CMP-pseudaminate production was associated with host immune pathways, particularly B- and T-cell signaling, and intestinal IgA production at WT-T2. Besides, the peptidoglycan biosynthesis V (β-lactam resistance) was identified as an upregulated microbial pathway at WT-T2. The β-lactam resistance occurs in but is not limited to *E. coli* through horizontal gene transfer (the dissemination of genetic mobile elements) [67, 68]. The observed pathway may indicate that STEC O157 may actively interact with mucosa-attached microbiota for genetic variability to improve its survival and colonization. Previous studies also identified that *Paeniclostridium* is capable of peptidoglycan biosynthesis [69, 70], and the similar functionality between STEC and *Paeniclostridium* may indicate the potential of *Paeniclostridium* interacting with STEC O157 during colonization**.** Similarly, both *E. coli* and *Paeniclostridium* are able to metabolize rhamnose [70, 71]. The rhamnose is a common component of the cell wall and essential for bacterial virulence and/or viability [72]. The degradation of the rhamnose pathway was upregulated at RE-T5 and associated with T-cells and the chemokine signaling pathway, suggesting that active rhamnose fermentation of *E. coli* and *Paeniclostridium* could be the intermediate for host–microbial interactions.

**Figure 8:**
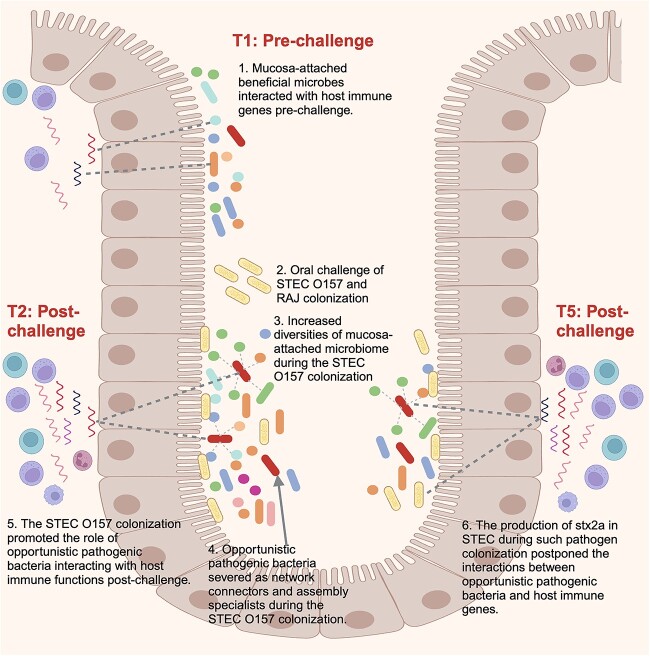
The proposed pathogen-mucosa–attached microbiome−host model for host–microbial interactions upon STEC O157 colonization in veal calves. The diagram was created using Biorender (www.biorender.com).

Taking these data together, we proposed a pathogen mucosa–attched commensals–host model in which the pathogen colonization initiates the shift of profiles, interactions, assembly, and functions of mucosa–attched microbiota, which further impacts host immune responses and differential host–microbial interactions. In our study, the colonization of STEC O157 first induced structural and functional variations of active mucosa-attached microbiome followed by altered microbial interactions and assembly patterns (Fig. 8). Through such processes, keystone opportunistic pathogens and predicted microbial pathways were identified to be associated with host immunity alterations and differed host−microbiome interactions (Fig. 8). We also noticed that stx2a expressions can be a factor affecting the pathogen–gut commensals–host model by postponing the occurrence of host–*Paeniclostridium* interactions and limiting interactions between gut commensals and host immune genes, highlighting the role of stx2a in shifting host–microbial interactions instead of affecting rectal mucosal microbial profiles. The proposed model can be useful to further our understanding of STEC O157 colonization mechanisms and may apply to other similar studies. Although this proposed model could improve our understanding of STEC O157 colonization, in the current study we did not evaluate long-term interactions between mucosa−attached microbes and the host, and whether the proposed model is relevant to changes in host age and species warrants further research. Besides, future studies are required to assess how mucosa−attached microbes could regulate interactions between pathogens and other host functions (i.e. gut barrier functions) and how virulence factors of STEC O157 (i.e. type III secretion system, adhesions) interact with mucosa-attached microbiota and host for facilitating pathogen colonization.

## Conclusion

Our comprehensive assessment of active mucosa-attached microbiota and their role during STEC O157 infection revealed their profiles, functions, and assembly patterns were Shiga toxin2 subtype dependent, which can also affect microbe–microbe and pathogen–host–commensal interactions. This study also showed the first evidence of opportunistic pathogenic taxa (*Paeniclostridium* and *Gallibacterium*) affecting microbial community assembly and interactions during pathogen colonization. We propose that STEC O157 indirectly affects the host through interactions with mucosa–attaached microbiota, particularly by regulating the relative abundance of *Paeniclostridium* and its interactions with other commensals and host immune functions, which warrant further study using in vivo and in vitro models. Taken together, our findings suggest that the strategy for mucosa–attached microbiota interacting with the host is subjected to pathogen colonization, of which interactions between mucosa-attached microbiota and the host could shift from beneficial microbes-driven under host homeostasis to opportunistic pathogenic microbes-driven after the pathogen colonization, promoting the understanding of the role of mucosa–attached microbiota for affecting pathogen–commensal–host interactions.

## Supplementary Material

Pan_et_al_Supp_figures_ISMEJ-D-24-00405_final_wrae127

Pan_et_al_Supp_Tables_SUB_ISMEJ-D-24-00405_final_wrae127

Pan_et_al_Supp_dataset_1_SUB_ISMEJ-D-24-00405_final_wrae127

Pan_et_al_Supp_dataset_2_SUB_SUB_ISMEJ-D-24-00405_final_wrae127

Pan_et_al_Supp_methods_SUB_ISMEJ-D-24-00405_R3_wrae127

## Data Availability

All sequence data have been deposited to NCBI Sequence Read Archive (SRA) under accession numbers PRJNA991158 (RNA sequencing) and PRJNA988112 (Amplicon sequencing).
