# Supplementary material for: Linking active rectal mucosa–attached microbiota to host immunity reveals its role in host–pathogenic STEC O157 interactions
Source: ISME J. 2024 Jul 10;18(1):wrae127. doi: 10.1093/ismejo/wrae127 (PMC11304501; doi:10.1093/ismejo/wrae127)
Supplement: Pan_et_al_Supp_figures_ISMEJ-D-24-00405_final_wrae127 [file pan_et_al_supp_figures_ismej-d-24-00405_final_wrae127.docx]

Linking active rectal mucosa-attached microbiota to host immunity reveals its role in host-pathogenic STEC O157 interactions

Zhe Pan^1^, Yanhong Chen^1^, Mi Zhou^1^, Tim A. McAllister^2^, Tom N. Mcneilly^3^, Le Luo Guan^1,4*^

1 Department of Agricultural, Food and Nutritional Science, University of Alberta, Edmonton, AB T6G 2P5, Canada

2 Agriculture and Agri-Food Canada, Lethbridge Research Centre, Lethbridge, AB T1J 4B1, Canada

3 Moredun Research Institute, Penicuik EH26 0PZ, United Kingdom

4 Faculty of Land and Food Systems, The University of British Columbia, Vancouver, BC V6T 1Z4, Canada

*Corresponding author: Dr. Le Luo Guan

Email: leluo.guan@ubc.ca Tel: +1 604-822-1225

Mail address: Faculty of Land and Food Systems, The University of British Columbia, Vancouver, BC V6T 1Z4, Canada


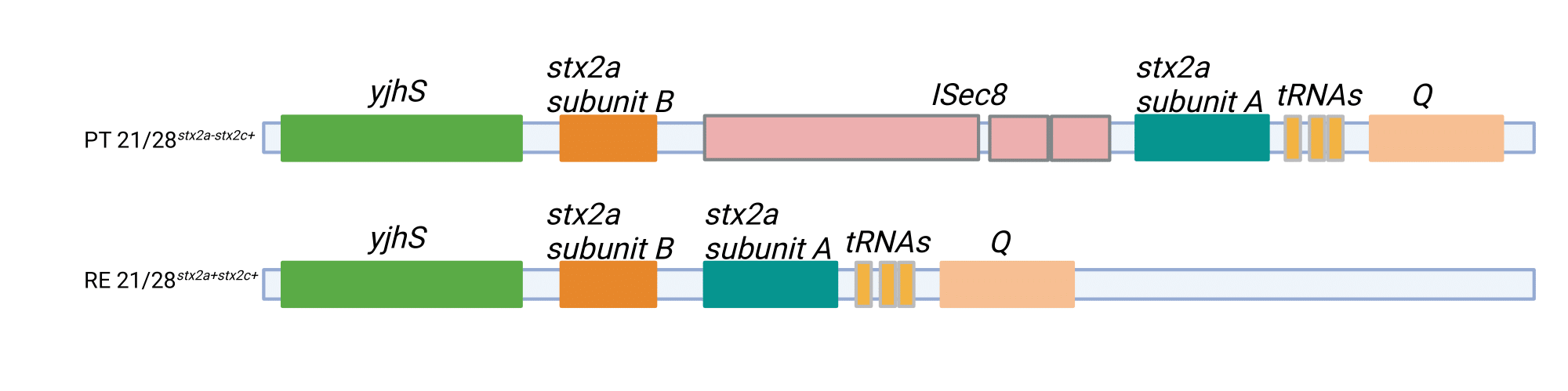


Fig S1. The structural differences between natural O157 (PT 21/28*^stx2a-stx2c^*^+^) and lab reconstructed O157 (RE 21/28 *^stx2a+stx2c+^*) strain. The RE 21/28 *^stx2a+stx2c+^* strain was constructed by removal of ISEc8 from stx2a in PT 21/28*^stx2a-stx2c^*^+^ in two steps by allelic exchange to ensure the production of functional stx2a and the deletion of ISEc8 was verified by PCR and sequencing.


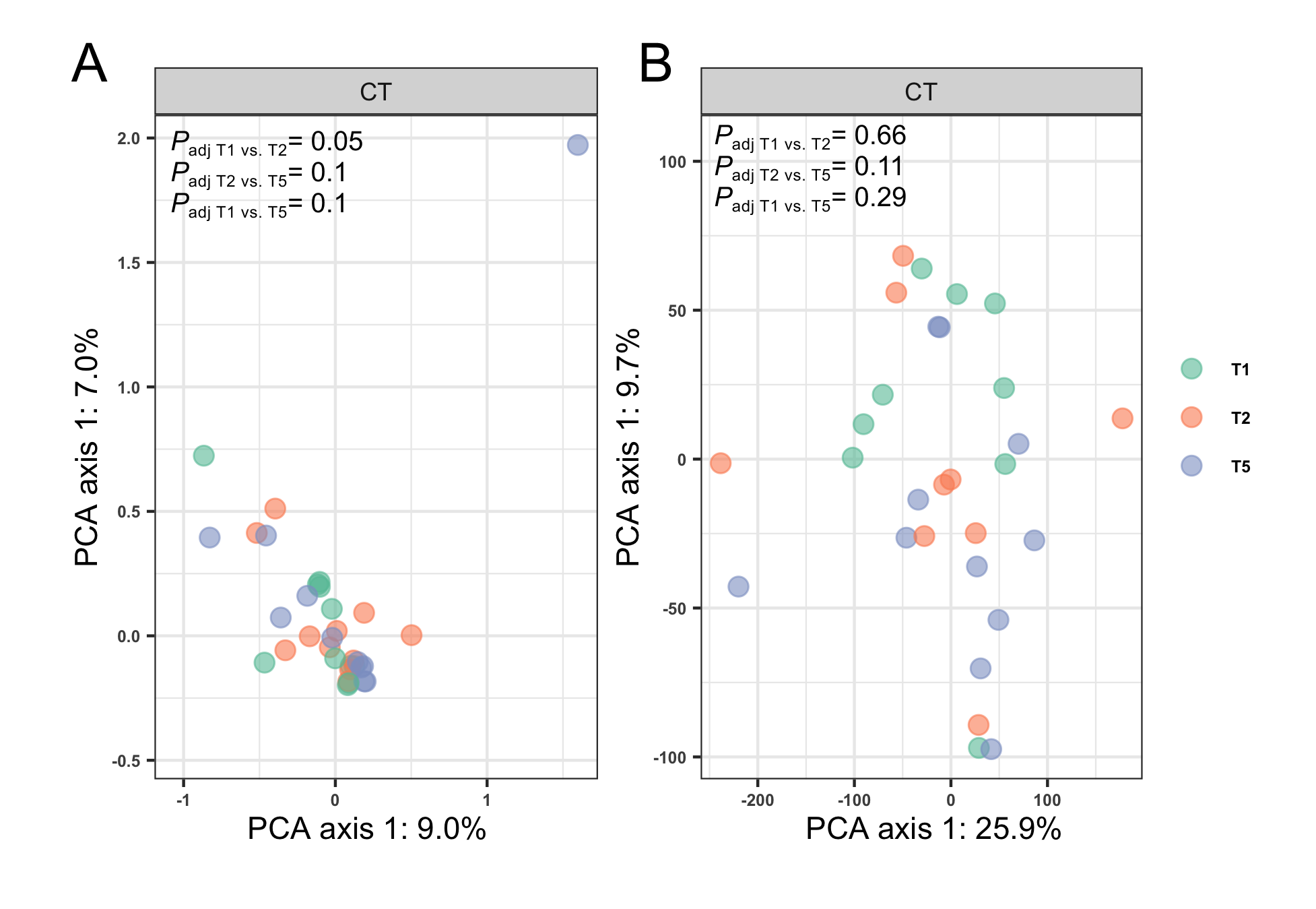
Fig S2. The principal component analysis (PCA) showing mucosa-attached microbiome (A) and host transcriptome (B) clustering patterns in calves without STEC O157 challenge. The pairwise PERMANOVA (Permutational analysis of variance) was adopted to test the significance of separation patterns across time for both mucosa-attached microbiome and host transcriptome (Benjamini-Hochberg adjusted *P* value <0.05 as a significance).


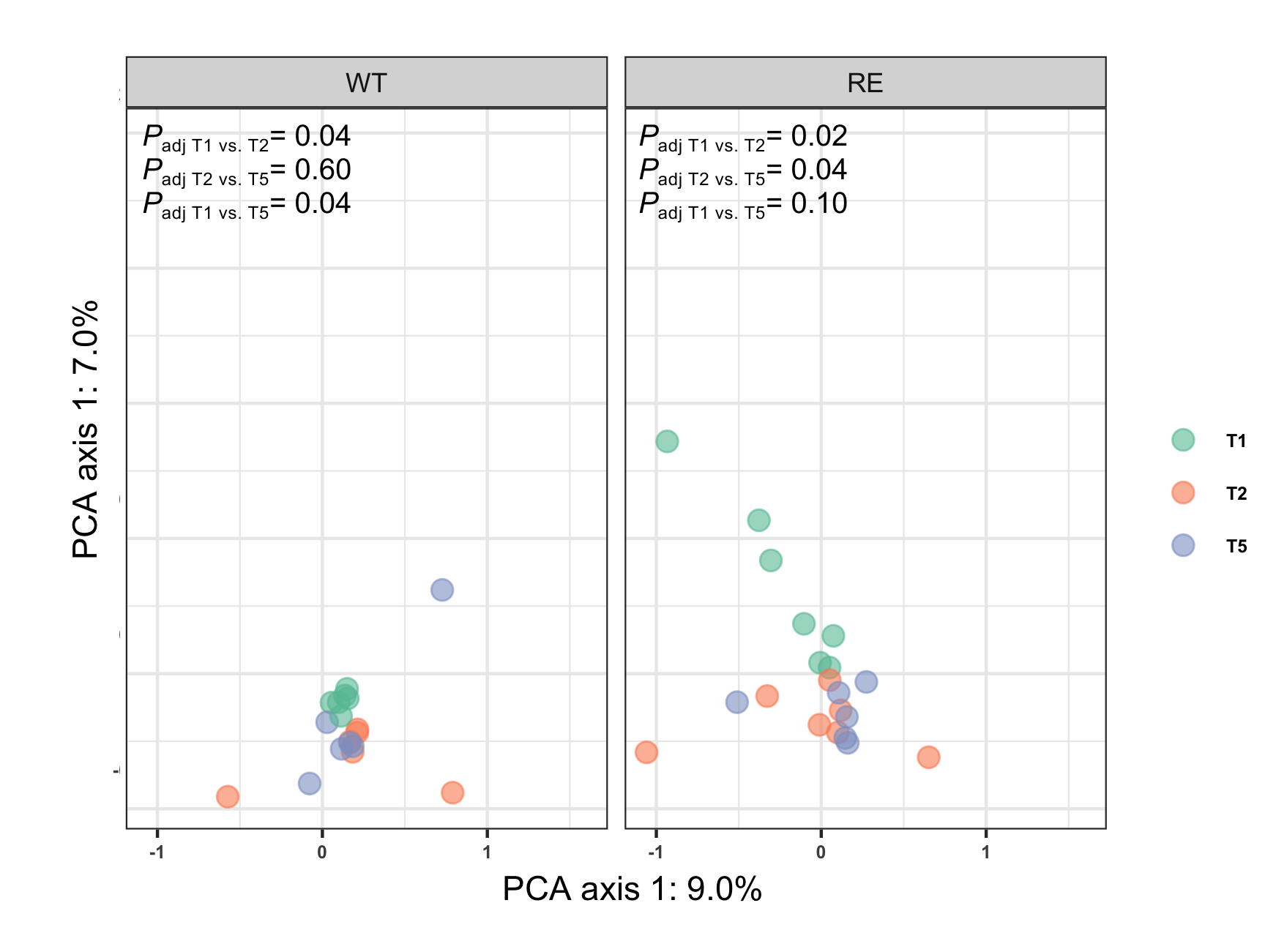
Fig S3. The principal component analysis (PCA) showing mucosa-attached microbiome clustering patterns for calves in the WT (left) and RE (right), respectively. The pairwise PERMANOVA (Permutational analysis of variance) was adopted to test the significance of separation patterns across time for mucosa-attached microbiome (Benjamini-Hochberg adjuste *P* value <0.05 as a significance).


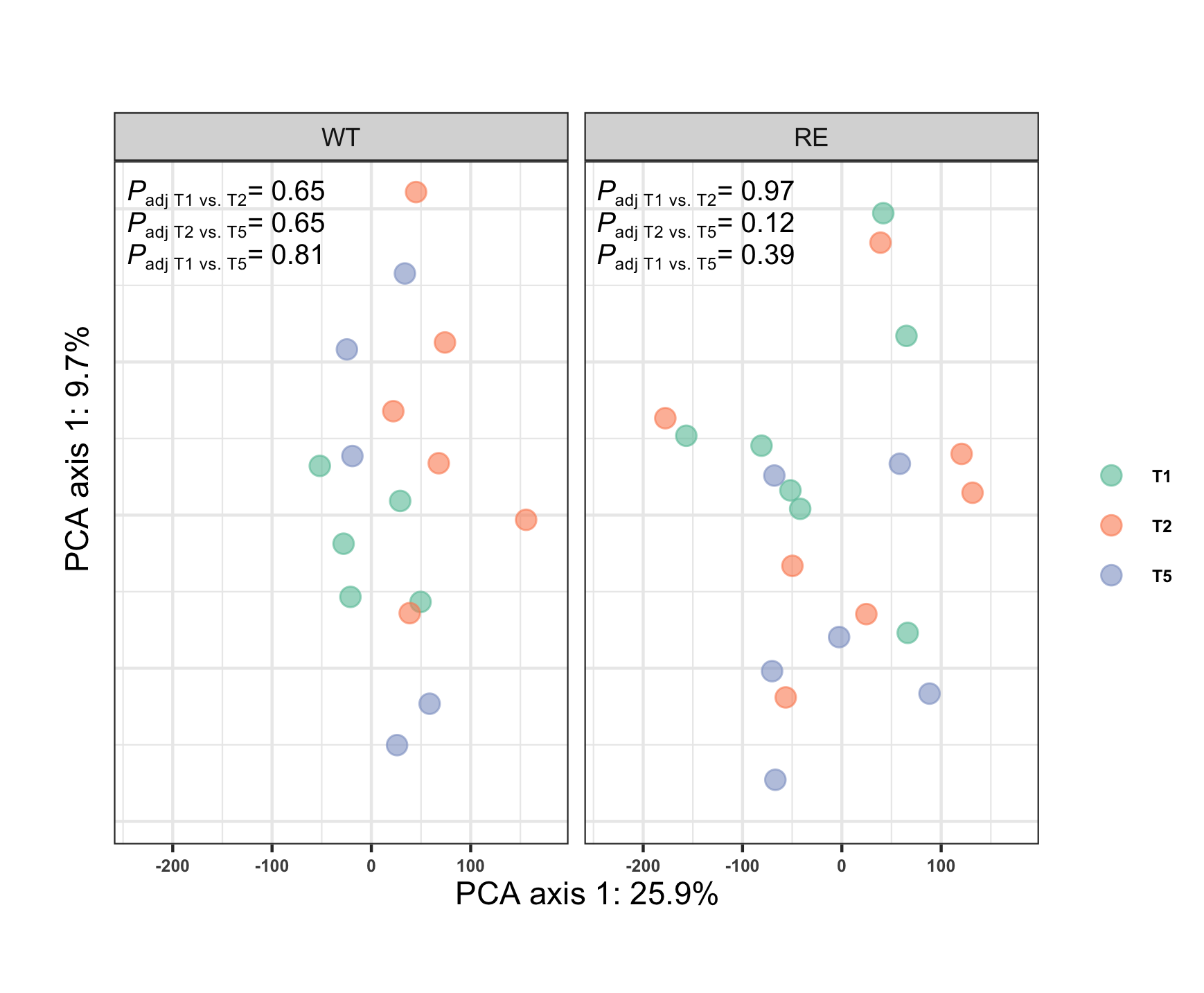
Fig S4. The principal component analysis (PCA) showing host transcriptome clustering patterns for calves in the WT (left) and RE (right), respectively. The pairwise PERMANOVA (Permutational analysis of variance) was adopted to test the significance of separation patterns across time for host transcriptome (Benjamini-Hochberg adjuste *P* value <0.05 as a significance).


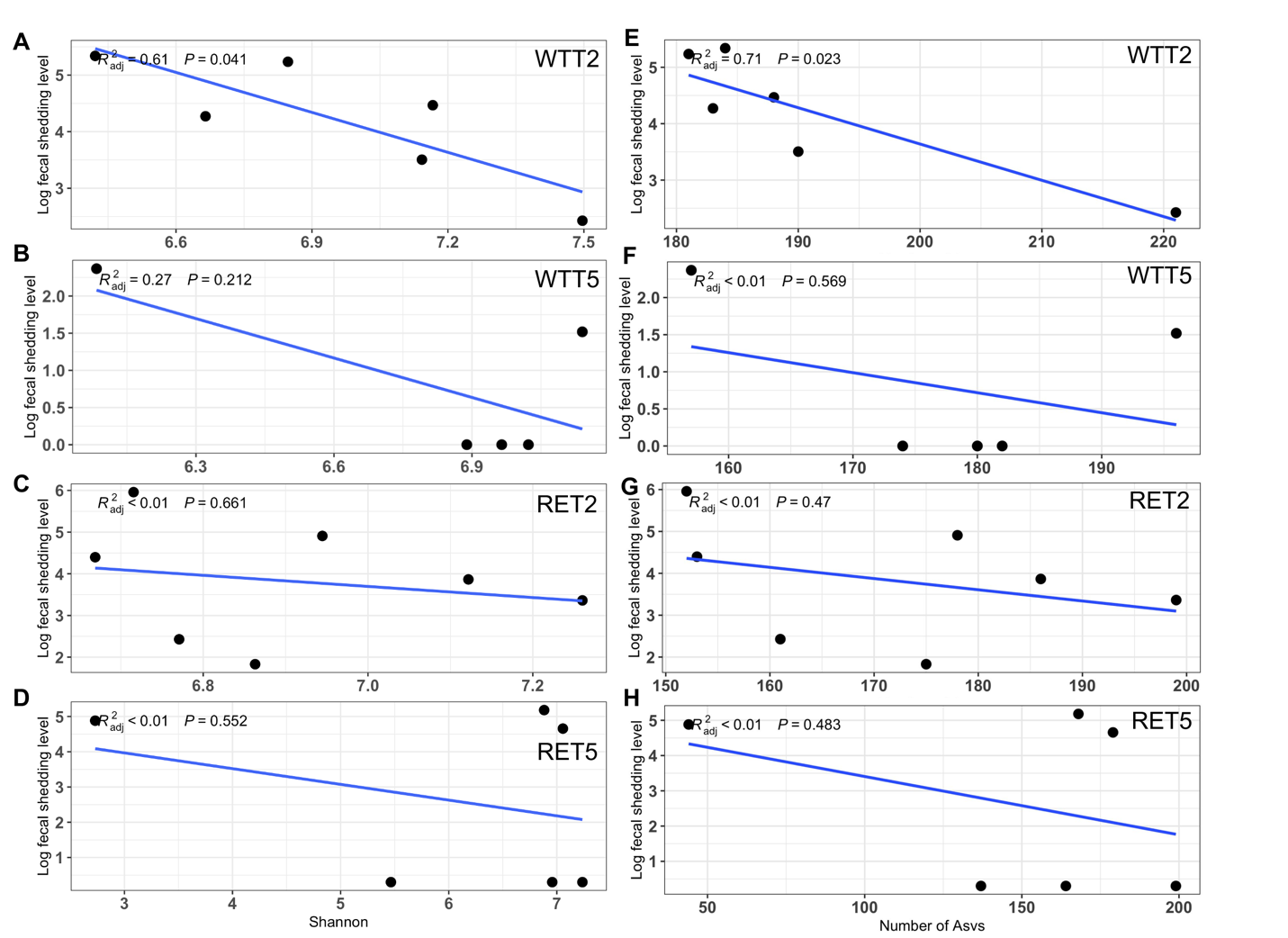


Fig S5. Relations between Shannon index(A-D) / Observed ASVs (E-H) index and log_10_ STEC O157 fecal shedding in calves in WT and RE groups at T2 and T5, respectively.


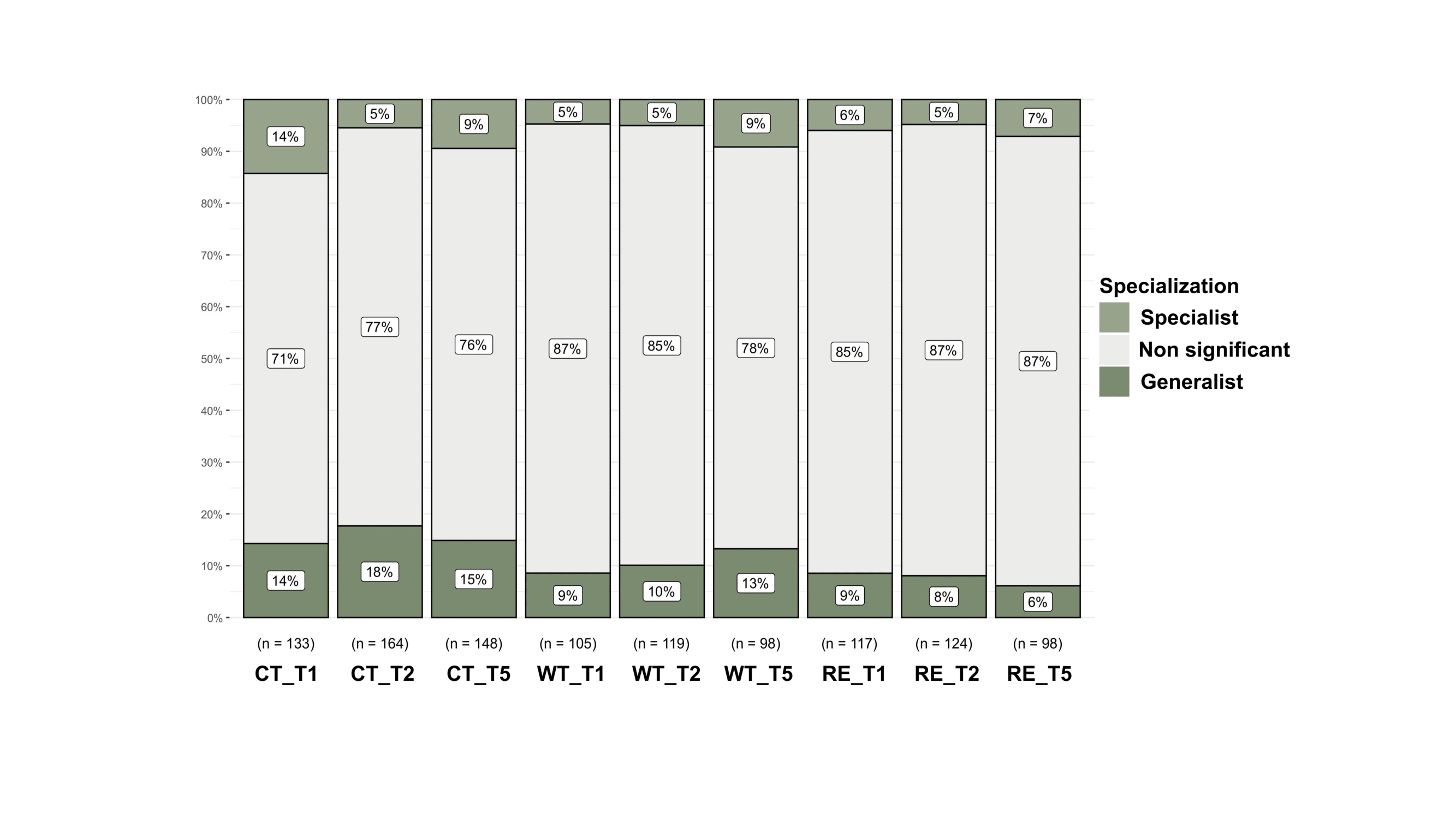
Fig S6. The microbial ecotype classification for CT, WT, and RE from T1 to T5. Three ecotypes (specialists, generalists, and non-significant as neutralists) were identified. The total number of microbes was labeled below each bar, and the percentage of each ecotype was labeled within each bar.


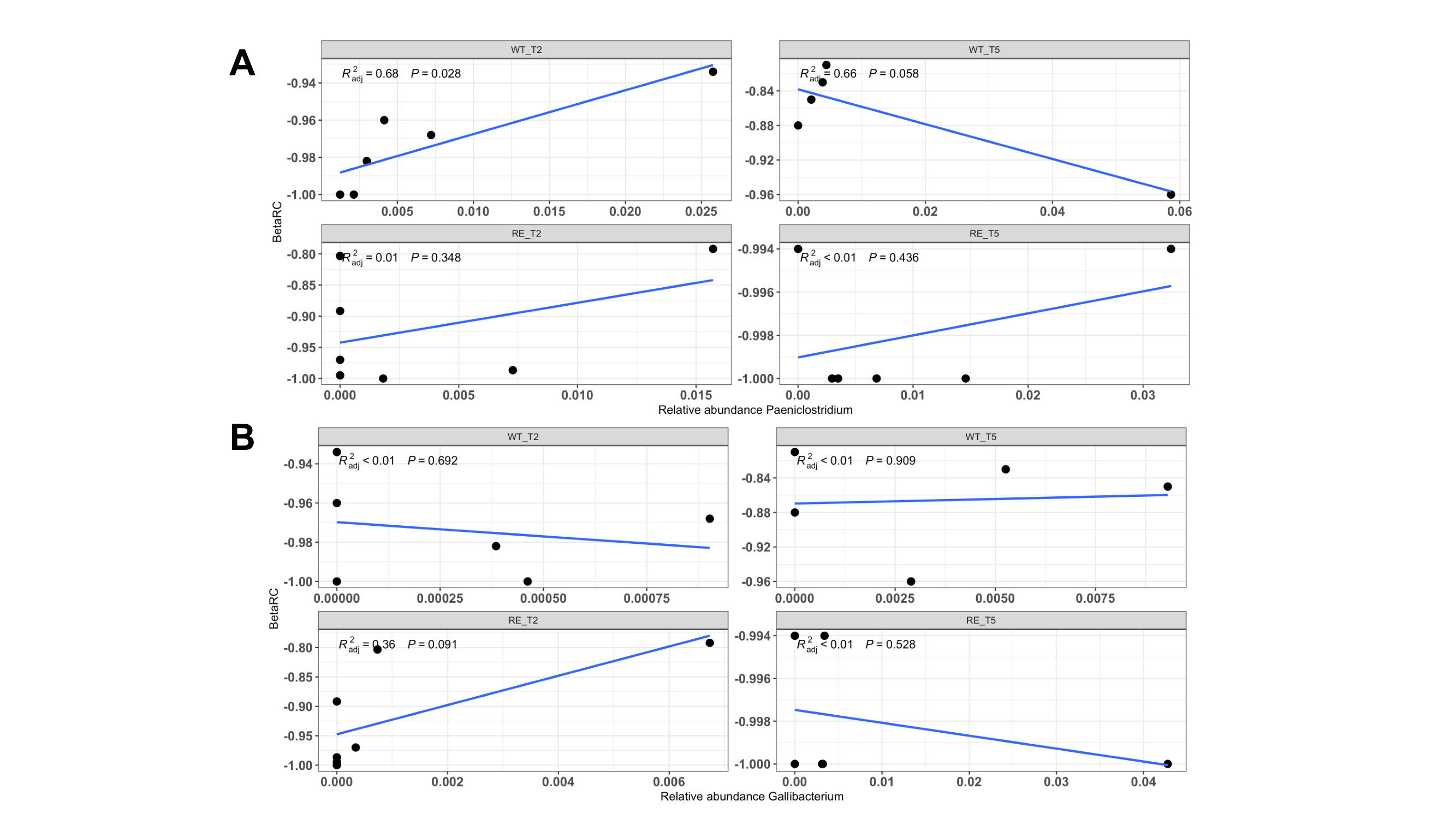


Fig S7. The linear regression models showing the relative abundance of *Paeniclostridium* (A) and *Gallibacterium* (B) and their relationships with Raup-Crick distance in WT (first and third row) and RE (second and fourth row) groups at T2 (left column) and T5 (right column).


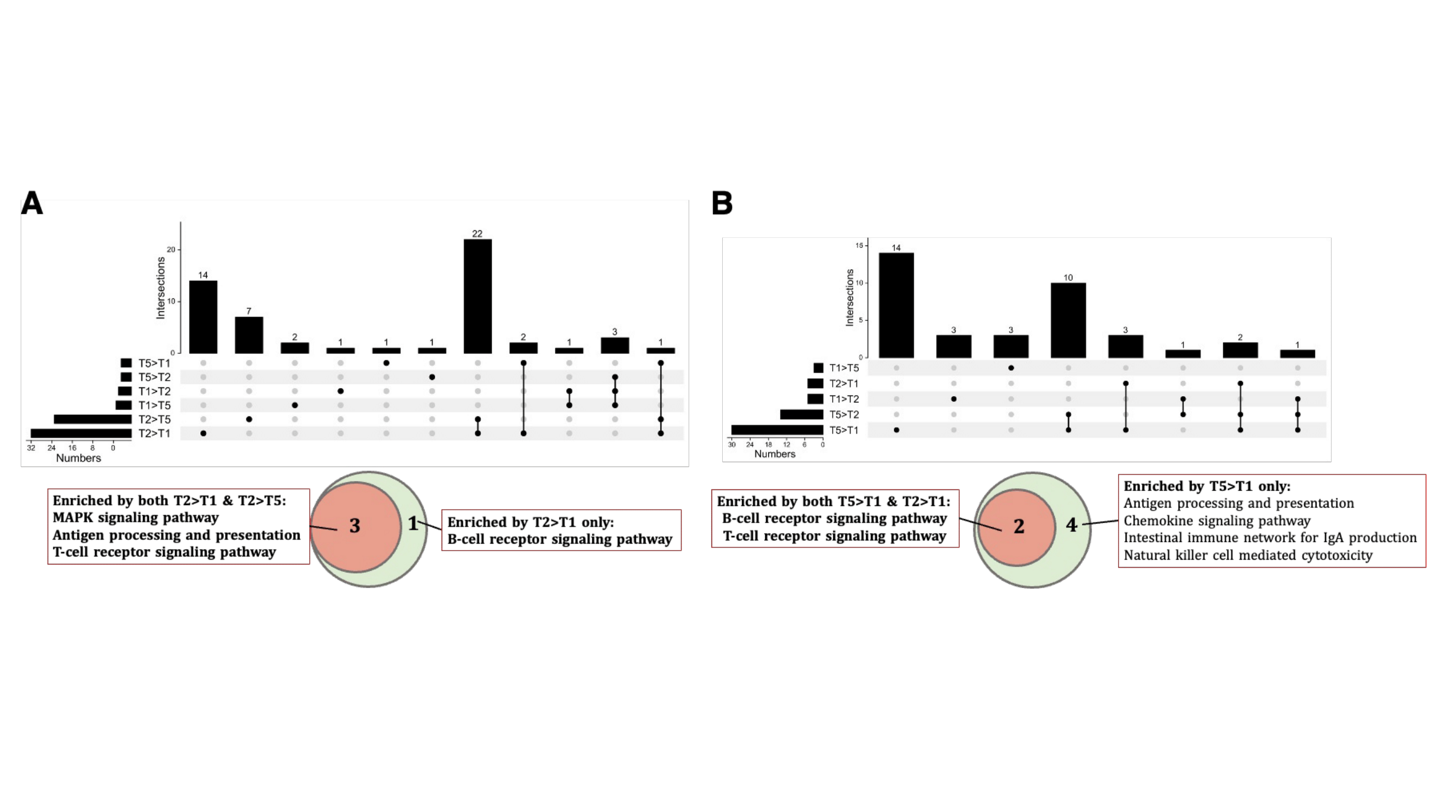


Fig S8. The upset plot shows intersections of host functional variations response to STEC O157 colonization in WT (A) and RE (B). The Venn plots below refer to intersections of host immune-related pathways. For example, T2>T1 refers to the host immune pathways that are upregulated in T2 compared to T1. Values on top of each column in the upset plot refer to the number of enriched pathways.


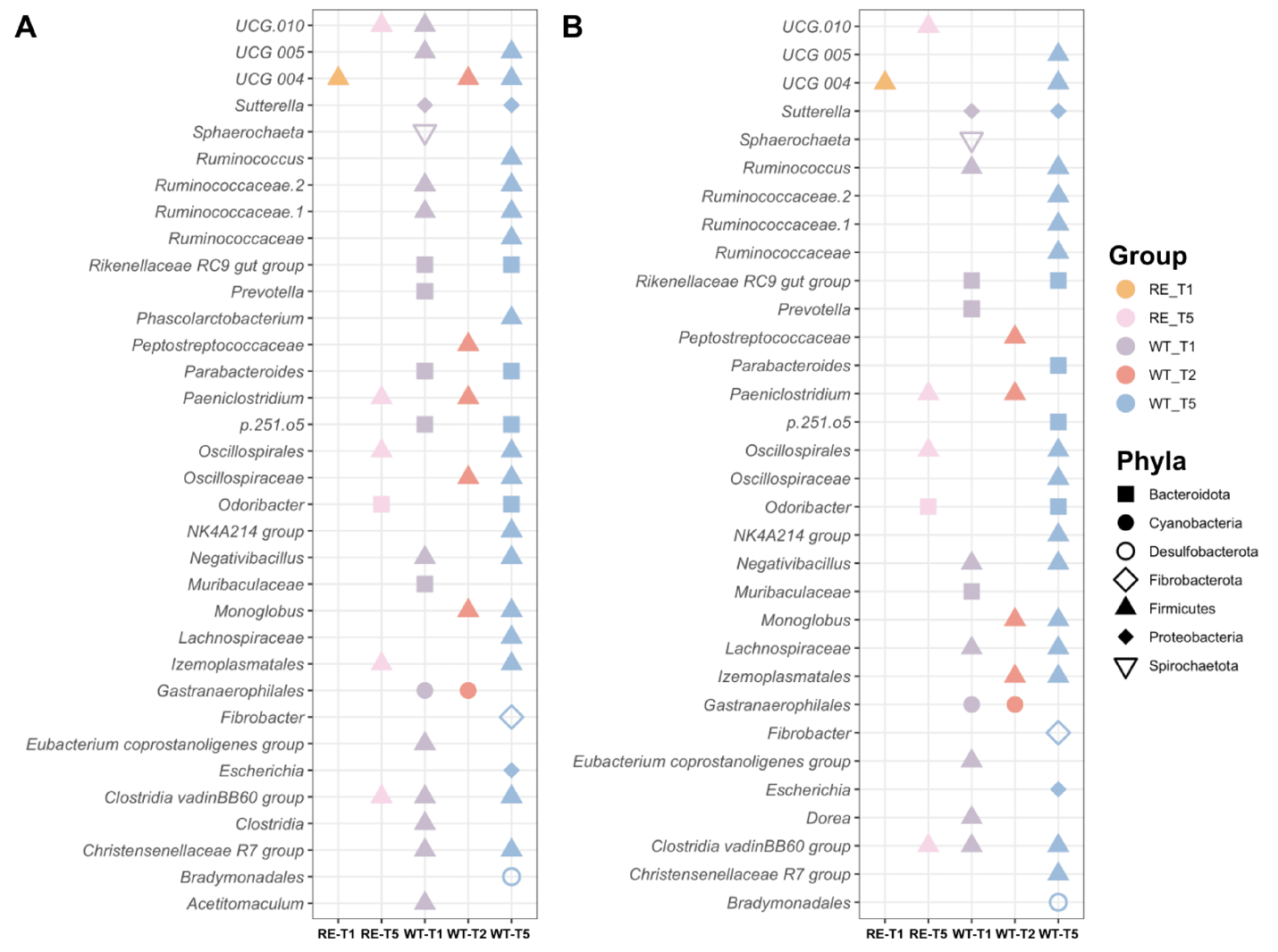


Fig S9. Significant interactions between host adaptive immune pathways and relative abundance of microbes from T1 to T5 among CT, WT, and RE groups using Spearman rank correlations (Absolute *R*>0.8 and *P* value<0.01 as a significance). A. T-cell receptor signaling pathway B. B-cell receptor signaling pathway


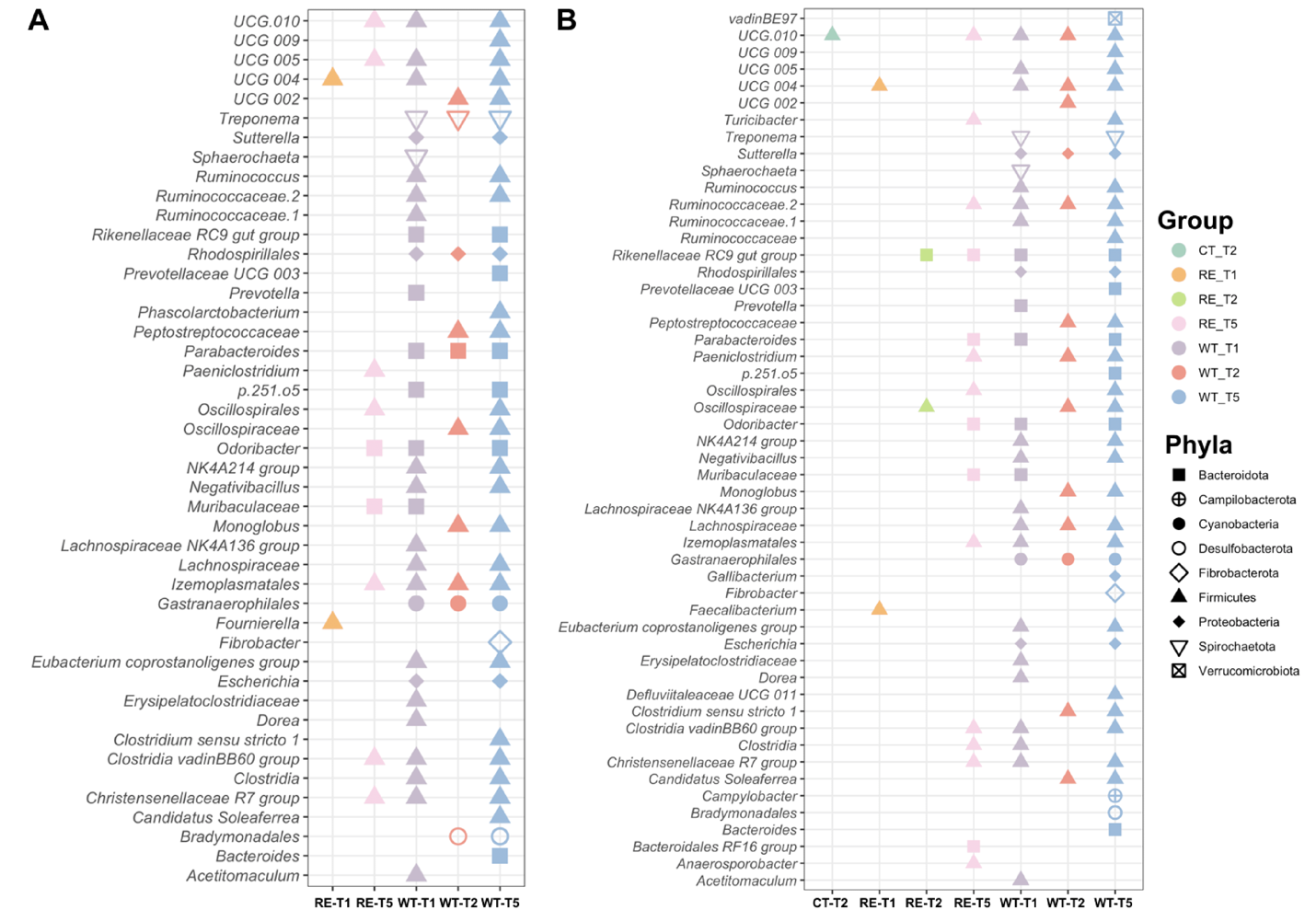
Fig S10. Significant interactions between chemokine signaling pathway (A), and MAPK signaling pathway (B), and relative abundance of microbes from T1 to T5 among CT, WT, and RE groups using Spearman rank correlations (Absolute *R*>0.8 and *P* value<0.01 as a significance).


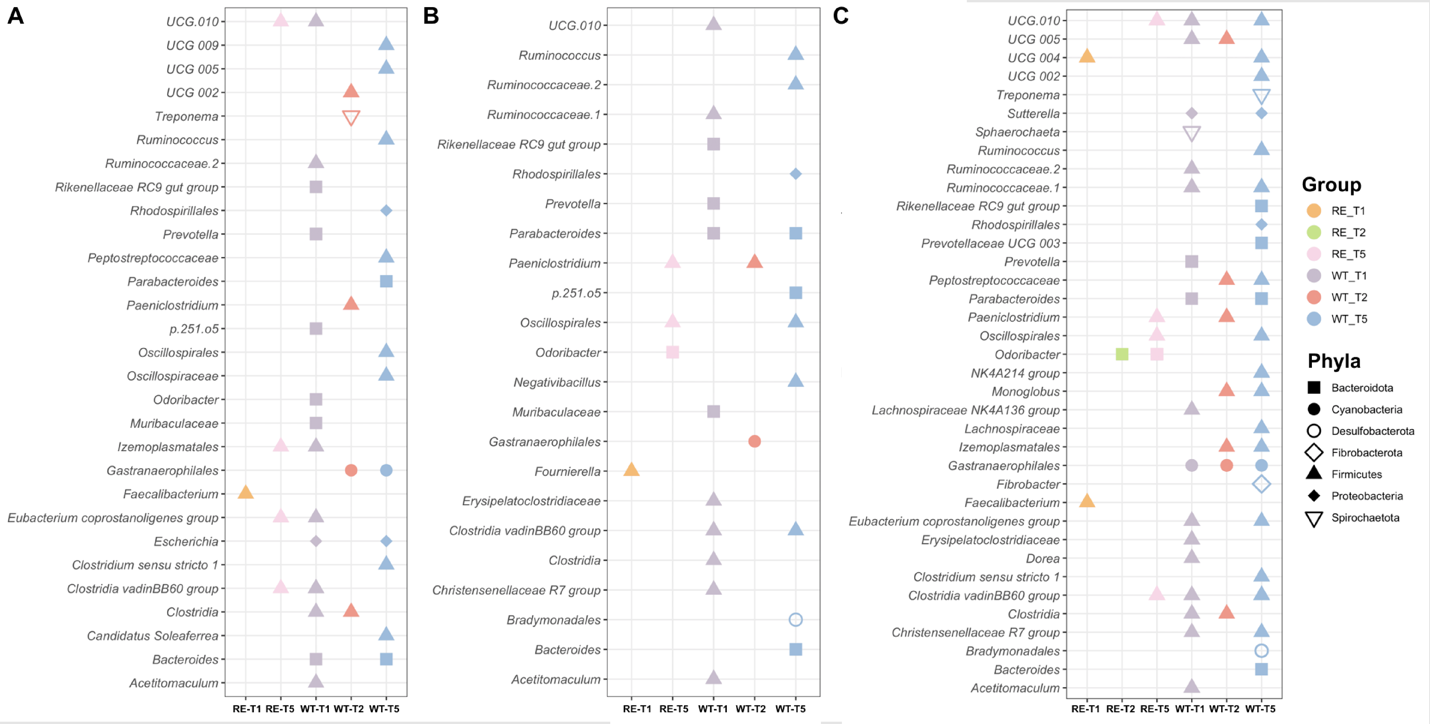


Fig S11. Significant interactions between antigen processing and presentation (A), intestinal immune network for IgA production (B), natural killer cell mediated cytotoxicity (C), and relative abundance of microbes from T1 to T5 among CT, WT, and RE groups using Spearman rank correlations (Absolute *R*>0.8 and *P* value<0.01 as a significance).


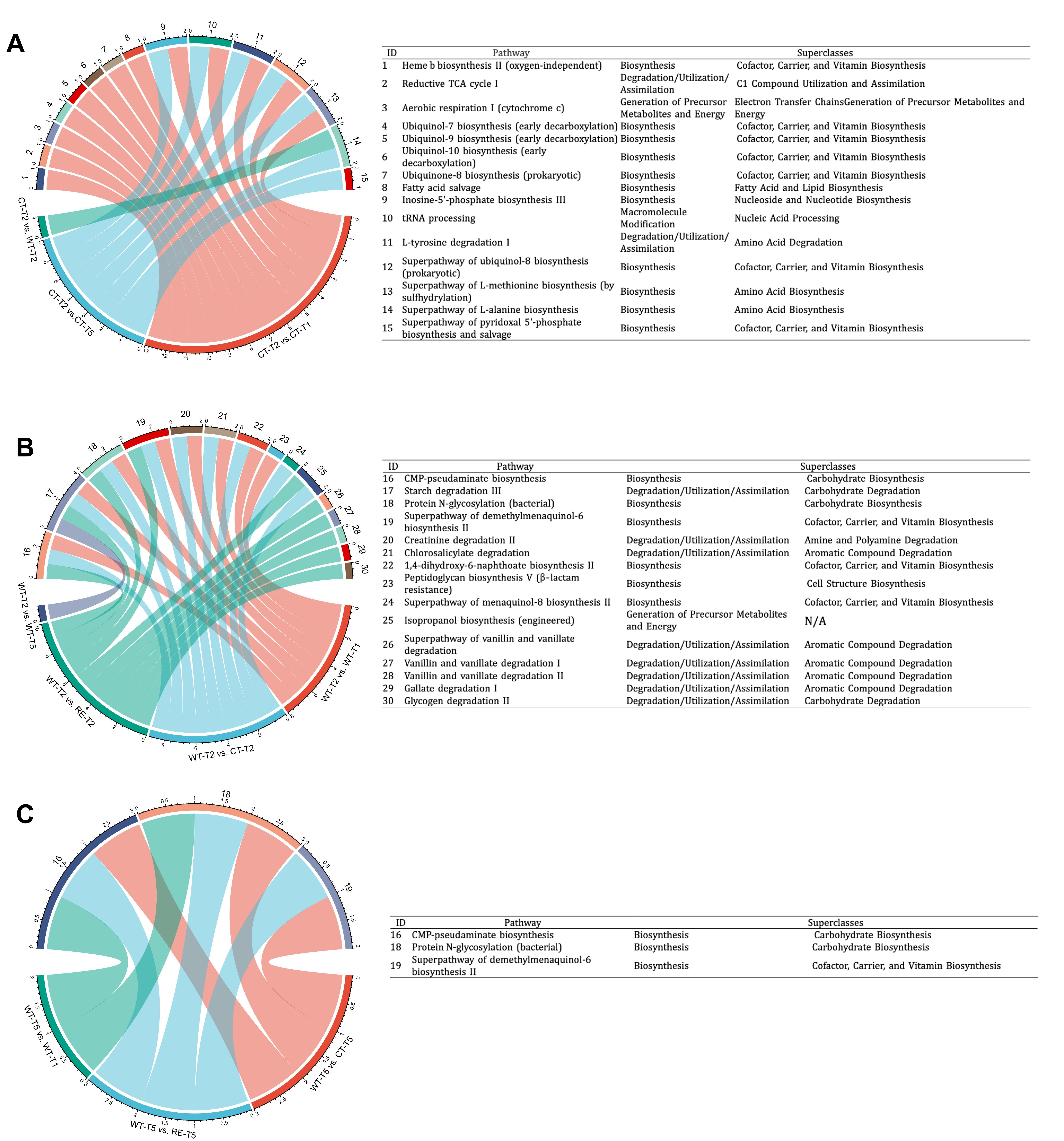
Fig S12. The circos plot showing altered microbial functions at CT-T2 (A), WT-T2 (B), and WT-T5 (C).


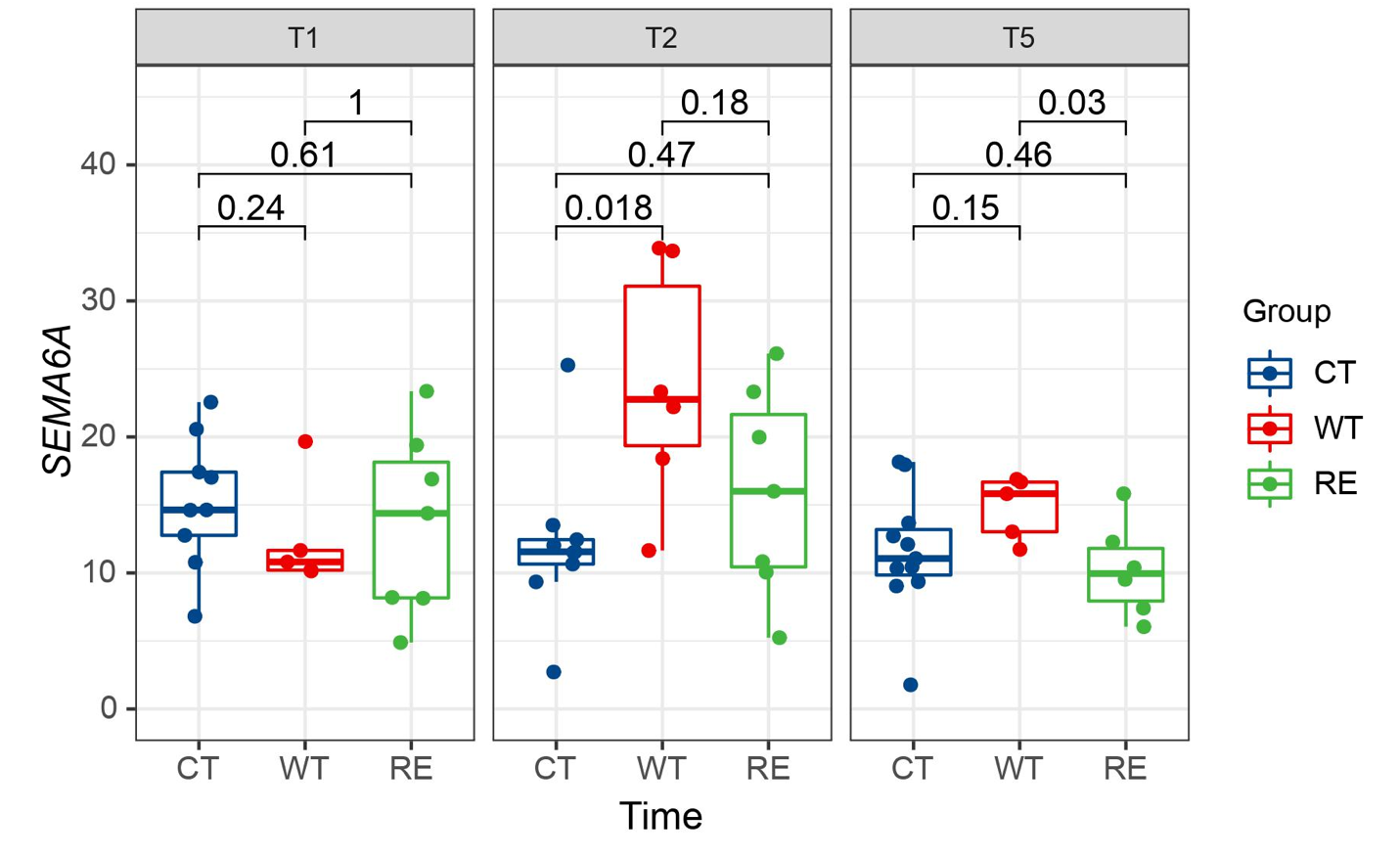
Fig S13. The comparison of the expression level of *SEMA6A* across each group from T1 to T5.
