# Supplementary material for: Linking active rectal mucosa–attached microbiota to host immunity reveals its role in host–pathogenic STEC O157 interactions
Source: ISME J. 2024 Jul 10;18(1):wrae127. doi: 10.1093/ismejo/wrae127 (PMC11304501; doi:10.1093/ismejo/wrae127)
Supplement: Pan_et_al_Supp_Tables_SUB_ISMEJ-D-24-00405_final_wrae127 [file pan_et_al_supp_tables_sub_ismej-d-24-00405_final_wrae127.docx]

Table S1. Primer used to fecal screening of stx1 and stx2 when calves entering the trial

| Primer used for fecal screening of stx1 and stx2 | |
| --- | --- |
| stx1-Forward | TTTGTYACTGTSACAGCWGAAGCYTTACG |
| stx1-Reverse | CCCCAGTTCARWGTRAGRTCMACRTC |
| Stx2-Forward | TTTGTYACTGTSACAGCWGAAGCYTTACG |
| stx2-Reverse | CCCCAGTTCARWGTRAGRTCMACRTC |

Table S2. The average relative abundance of bacterial phylum across each group. The PROC MIXED models was used to determine factors affecting the differences of bacterial phylum across each group (*P* < 0.05 as significant).

| Group | CT | | | WT | | | RE | | | P | | |
| --- | --- | --- | --- | --- | --- | --- | --- | --- | --- | --- | --- | --- |
| Time | T1 | T2 | T5 | T1 | T2 | T5 | T1 | T2 | T5 | Group | Time | Group*Time |
| *Actinobacteriota* | 0.0081  ±0.0192 | 0.0136  ±0.0348 | 0.0065  ±0.0196 | 0.0053  ±0.0060 | 0.0149  ±0.0175 | 0.0002  ±0.0005 | 0.0004  ±0.0009 | 0.0114  ±0.0178 | 0.0005  ±0.0008 | 0.15 | 0.01* | 0.02* |
| *Bacteroidota* | 0.0502  ±0.0337 | 0.0502  ±0.0328 | 0.0823  ±0.0402 | 0.0370  ±0.0256 | 0.0573  ±0.0281 | 0.0306  ±0.0279 | 0.0735  ±0.0573 | 0.0699  ±0.0300 | 0.0698  ±0.0452 | 0.03* | 0.51 | 0.04* |
| *Firmicutes* | 0.8265  ±0.1078 | 0.6851  ±0.3084 | 0.7383  ±0.2057 | 0.8163  ±0.0766 | 0.6935  ±0.0871 | 0.7982  ±0.1095 | 0.7339  ±0.2323 | 0.7664  ±0.1457 | 0.7958  ±0.1675 | 0.56 | 0.31 | 0.67 |
| *Proteobacteria* | 0.0780  ±0.1013 | 0.1913  ±0.3262 | 0.1399  ±0.1686 | 0.03980  ±0.0294 | 0.1756  ±0.0967 | 0.0398  ±0.0312 | 0.1542  ±0.2389 | 0.1164  ±0.0960 | 0.1049  ±0.1567 | 0.71 | 0.35 | 0.45 |

| Group | Week | Nodes | Edges | Modularity | Average  degree | Clustering  coefficient |
| --- | --- | --- | --- | --- | --- | --- |
| CT | T1 | 132 | 775 | 0.46 | 11.74 | 0.56 |
|  | T2 | 163 | 906 | 0.55 | 11.12 | 0.72 |
|  | T5 | 144 | 1173 | 0.49 | 16.29 | 0.66 |
| WT | T1 | 105 | 300 | 0.73 | 5.71 | 0.80 |
|  | T2 | 117 | 494 | 0.75 | 8.44 | 0.83 |
|  | T5 | 97 | 261 | 0.72 | 5.38 | 0.63 |
| RE | T1 | 116 | 550 | 0.52 | 9.48 | 0.67 |
|  | T2 | 122 | 642 | 0.54 | 10.52 | 0.73 |
|  | T5 | 95 | 353 | 0.69 | 7.43 | 0.70 |

Table S3. The network topological properties of microbial interactions among rectal mucosa microbial communities among three groups.

Table S4. The attributions of microbial genera based on their network roles.

|  | **Group** | **Week** | | | ***χ*^2^ test** | |
| --- | --- | --- | --- | --- | --- | --- |
|  |  | **T1** | **T2** | **T5** | ***χ*^2^** | ***p*** |
| Connectors | CT | 0 | 4 | 3 | N/A | 0.45 |
|  | WT | 4 | 3 | 3 |  |  |
|  | RE | 2 | 3 | 1 |  |  |
| Peripherals | CT | 132 | 160 | 142 | 2.1 | 0.71 |
|  | WT | 101 | 114 | 94 |  |  |
|  | RE | 114 | 120 | 94 |  |  |

Chi-square test was used to test the equality of numbers of specialized microbes. Fisher’s exact test was used as the count of one cell was below 5.

Table S5. The Chi-square test analyzes the equality of proportions of assembly process belonging to deterministic- or stochastic driven (P<0.05 as a significance).

| Group | Raup-Crick beta diversity | | | |
| --- | --- | --- | --- | --- |
|  | Deterministic (%) | Stochastic (%) | χ^2^ | *P* value |
| All group | 73 | 27 | 54.84 | 0.01* |
| CT-T1 | 67 | 33 | 6.56 | 0.01* |
| CT-T2 | 47 | 53 | 0.16 | 0.69 |
| CT-T5 | 82 | 18 | 22.27 | 0.01* |
| WT-T1 | 93 | 7 | 0.27 | 0.01* |
| WT-T2 | 80 | 20 | 5.40 | 0.02* |
| WT-T5 | 67 | 33 | 1.67 | 0.20 |
| RE-T1 | 86 | 14 | 10.71 | 0.01* |
| RE-T2 | 81 | 19 | 8.05 | 0.01* |
| RE-T5 | 100 | 0 | 15.00 | 0.01* |

Table S6. The quantity of specialized microbes among microbial community assembly in CT, WT and RE groups from T1 to T5. Chi-square test was used to test the equality of numbers of specialized microbes.

| Attributes | Group | Week | | | | χ^2^ test | |
| --- | --- | --- | --- | --- | --- | --- | --- |
|  |  | T1 | T2 | T5 | χ^2^ | | *P* |
| Generalists | CT | 19 | 29 | 22 | 2.2 | | 0.7 |
|  | WT | 9 | 12 | 13 |  |  |  |
|  | RE | 10 | 10 | 6 |  |  |  |
| Non-significant | CT | 95 | 126 | 112 | 3.7 | | 0.4 |
|  | WT | 91 | 101 | 76 |  |  |  |
|  | RE | 100 | 108 | 85 |  |  |  |
| Specialists | CT | 19 | 9 | 14 | 2.6 | | 0.6 |
|  | WT | 5 | 6 | 9 |  |  |  |
|  | RE | 7 | 6 | 7 |  |  |  |

Table S7. Host immune related pathways identified from the gene set enrichment analysis.

|  | Expressed genes^1^ | Total genes involved in each pathway^2^ | Percentage of expressed genes/ total genes in each pathway |
| --- | --- | --- | --- |
| Antigen processing and presentation | 43 | 88 | 0.49 |
| Chemokine signaling pathway | 171 | 189 | 0.9 |
| Intestinal immune network for IgA production | 32 | 48 | 0.67 |
| Natural killer cell-mediated cytotoxicity | 95 | 137 | 0.7 |
| MAPK signaling pathway | 259 | 267 | 0.97 |
| T cell receptor signaling pathway | 107 | 108 | 0.99 |
| B cell receptor signaling pathway | 73 | 75 | 0.97 |

1 The number of genes expressed within each pathway among all samples

2 The number of genes belonging to each pathway

Table S8. Altered microbial pathways at RE-T5 compared to that at CT-T5.

| Upregulated pathways (RE-T5 vs. CT-T5) | Superclasses | |
| --- | --- | --- |
| Superpathway of N-acetylglucosamine, N-acetylmannosamine and N-acetylneuraminate degradation | Degradation/Utilization/Assimilation | Amine and Polyamine Degradation Superpathways |
| 2-methylcitrate cycle II | Degradation/Utilization/Assimilation | Carboxylate Degradation |
| 2-methylcitrate cycle I | Degradation/Utilization/Assimilation | Carboxylate Degradation |
| Superpathway of fucose and rhamnose degradation | N/A | |
| Superpathway of glycol metabolism and degradation | Degradation/Utilization/Assimilation | Alcohol Degradation |
| Allantoin degradation to glyoxylate III | Degradation/Utilization/Assimilation | Amine and Polyamine Degradation |
| D-galactarate degradation I | Degradation/Utilization/Assimilation | Carboxylate Degradation |
| D-glucarate degradation I | Degradation/Utilization/Assimilation | Carboxylate Degradation |
| Superpathway of D-glucarate and D-galactarate degradation | N/A | |
| TCA cycle IV (2-oxoglutarate decarboxylase) | Generation of Precursor Metabolites and Energy | TCA cycle |
| Superpathway of glyoxylate bypass and TCA | N/A | |
