# Supplementary material for: Linking active rectal mucosa–attached microbiota to host immunity reveals its role in host–pathogenic STEC O157 interactions
Source: ISME J. 2024 Jul 10;18(1):wrae127. doi: 10.1093/ismejo/wrae127 (PMC11304501; doi:10.1093/ismejo/wrae127)
Supplement: Pan_et_al_Supp_methods_SUB_ISMEJ-D-24-00405_R3_wrae127 [file pan_et_al_supp_methods_sub_ismej-d-24-00405_r3_wrae127.docx]

Linking active rectal mucosa-attached microbiota to host immunity reveals its role in host-pathogenic STEC O157 interactions

Zhe Pan^1^, Yanhong Chen^1^, Mi Zhou^1^, Tim A. McAllister^2^, Tom N. Mcneilly^3^, Le Luo Guan^1,4*^

1 Department of Agricultural, Food and Nutritional Science, University of Alberta, Edmonton, AB, Canada

2 Agriculture and Agri-Food Canada, Lethbridge Research Centre, Lethbridge, AB, Canada

3 Moredun Research Institute, Penicuik, United Kingdom

4 Faculty of Land and Food Systems, The University of British Columbia, Vancouver, British Columbia, Canada

*Corresponding author

Email: leluo.guan@ubc.ca Tel: +1 604-822-1225

Running title: active rectal mucosa attached microbiota affects host-pathogen interactions

**Supplementary Methods**

1. Fecal samples were collected from the rectum daily for the first 18 days post oral challenge and then every other day for challenged calves. For unchallenged calves, fecal samples were taken every other day. Ten grams of feces were placed into 90 ml of sterile PBS and 10-fold serial dilutions were then prepared in PBS and 100 μl from three dilutions across a 1,000-fold range from 10^3^ to 10^6^ were plated onto sorbitol MacConkey agar plates containing 15 μg nalidixic acid (NAL-SMAC). Plates were incubated at 37°C overnight and colonies were counted on the most suitable dilution plates. Randomly selected colonies from each plate were confirmed as O157 positive by using a latex agglutination kit (Oxoid, Basingstoke, United Kingdom) following the manufacturer’s instructions. Samples from the control animals were plated out directly onto cefixime –tellurite (CT)-SMAC plates containing 0.05 mg/L cefixime, 2.5 mg/L tellurite; incubated overnight at 37°C and then enumerated the next day.

2. The RNA was isolated from 0.1 g biopsy tissue using trizol reagent (Invitrogen Corporation, Carlsbad, CA, USA), purified using the RNeasy MinElute Cleanup kit (Qiagen, Valencia, CA, USA), and assessed using Agilent 2200 TapeStation (Agilent Technologies, Santa Clara, CA, USA) and Qubit 3.0 Fluorometer (Invitrogen, Carlsbad, CA, USA) with RIN number ≥ 7 and concentration ≥ 200 ng/μl. Total RNA (0.1 µg) was further subjected to reverse transcription to synthesize cDNA using a cDNA Synthesis Kit (Bio-Rad, Hercules, CA, USA). Single-stranded cDNA was amplified using Oligo(dT)12-18 (Life Technologies, Carlsbad, CA, USA) and SuperScript™ II RT (Life Technologies, Carlsbad, CA, USA) was used to synthesize double-strand cDNA.

To generate the mucosa-attached active bacterial compositional profiles, the bacterial V1-V3 region of the 16S rRNA was amplified from generated cDNA using bacterial primers Ba9F (5'-GAGTTTGATCMTGGCTCAG-3') and Ba515Rmod1 (5'-CCGCGGCKGCTGGCAC-3'). The PCR amplification products were verified using agarose gel (2%) electrophoresis and purified with a Qiagen Gel Extraction Kit (Qiagen, Germany). All amplicon libraries were sequenced using the MiSeq PE 300 platform (2 × 300 pair-end, Illumina) at Génome Québec, McGill University (Quebec, Canada).

Extracted total RNA (1 µg) was used for library construction using the Truseq Stranded Total RNA Sample Preparation kit (Illumina, San Diego, CA, USA) following the manufacturer’s instructions. The quality of constructed libraries was assessed using Agilent 2200 TapeStation and a Qubit 2.0 Fluorometer. RNA sequencing was performed using a HiSeq 4000 sequencing system (Illumina), with paired-end (100 bp) sequencing at Genome Quebec Innovation Centre, Montreal, Quebec, Canada.

3. Niche breadth refers to the diversity of recourses used by an individual (or species) within a certain environment [1], which can be measured:

$$B_{j}=\frac{1}{\sum_{i=1}^{N} P_{ij}^{2}}$$

Here, $B_{j}$ stands for niche breadth, $P_{ij}$ refers to the proportion of any species $i$ in a given sample, $j$ and $N$ is the total number of samples [2]. Niche breadth refers to the diversity of recourses used by an individual (or species) within a certain environment [1].

The microbial ecotypes quantify the role of each microbe within the microbial community by estimating the availability of resources to individuals [1]. The microbial ecotypes (generalist, specialist, neutralists) were classified based on the 1000-time quasiswap permutation simulations of niche breadth index using EcolUtils R package. The empirical niche breadth value of certain taxa that exceeded the 95% confidence interval of the null distribution was designated as a generalist, whereas those that were below the 95% confidence interval were defined as a specialist. Taxa that were within the 95% confidence interval was defined as neutralists [3]. When available recourses are limited, niche breadth is usually increased for species to gain more resources for survival and such taxa can occupy a broader niche and are defined as generalists. In contrast, species that selectively use specific resources and have a narrower niche breadth are defined as specialists [1].

4. For host transcriptomics using RNA sequencing, one sample from WT-T1, two samples from CT-T2, two samples from CT-T2, one sample from WT-T5, and one sample from RE-T5 were omitted due to the low RNA integrity number (RIN < 7). RNA-sequencing reads were first subjected to the quality filter and adapter trimming using FastQC and bbDuk. Filtered reads were then mapped against the reference bovine reference assembly ARS-UCD 1.2.99 using STAR (Version 2.7.1a). Feature counts were then generated using subread (Version 2.0.0) and were then normalized into TPM (transcripts per million).

The gene set enrichment analysis (GSEA) was used to identify varied pathways in response to the STEC O157 challenge in both WT and RE groups using R (altered pathways: absolute normalized enrichment score >1, nominal *P* value <0.05, FDR *q* value <0.1). **The GSEA** is a computational method that determines whether an *a priori* defined set of genes shows statistically significant. Particularly, the GSEA is based on *a priori* defined sets of genes, which are collections of genes that are functionally related or belong to the same biological pathway and to assess if gene sets representing certain biological functions are enriched or decreased under phenotype differences or experimental conditions [4, 5]. The GSEA assesses whether most genes within a specific set were fall at the extremes of the gene set, with the top and bottom representing the greatest expression variances. If the gene set falls at either the top (over-expressed) or bottom (under-expressed), the relevant biological pathway is thought to be up-regulated or down-regulated, respectively, likely linked to the observed phenotypic differences (referred to as the O157 challenge in our study) [5, 6]. The gene set database is Molecular Signatures Database (MSigDB), which is a resource of tens of thousands of annotated gene sets for use with GSEA, providing robust annotation of host biological pathways [5]. Hence, the GSEA considered all genes as background and is capable of identifying whether a biological pathway is up-/down- regulated [5, 6].

**References:**

1. Carscadden KA, Emery NC, Arnillas CA, Cadotte MW, Afkhami ME, Gravel D, et al. Niche Breadth: Causes and Consequences for Ecology, Evolution, and Conservation. *Q Rev Biology* 2020; **95**: 179–214. https://doi.org/10.1086/710388

2. Levins R. Evolution in Changing Environments. Princeton University Press.

3. Wu W, Logares R, Huang B, Hsieh C. Abundant and rare picoeukaryotic sub‐communities present contrasting patterns in the epipelagic waters of marginal seas in the northwestern Pacific Ocean. *Environ Microbiol* 2017; **19**: 287–300. https://doi.org/10.1111/1462-2920.13606

4. Mootha VK, Lindgren CM, Eriksson K-F, Subramanian A, Sihag S, Lehar J, et al. PGC-1α-responsive genes involved in oxidative phosphorylation are coordinately downregulated in human diabetes. *Nat Genet* 2003; **34**: 267–273. https://doi.org/10.1038/ng1180

5. Subramanian A, Tamayo P, Mootha VK, Mukherjee S, Ebert BL, Gillette MA, et al. Gene set enrichment analysis: A knowledge-based approach for interpreting genome-wide expression profiles. *Proc Natl Acad Sci* USA 2005; **102**: 15545–15550. https://doi.org/10.1073/pnas.0506580102

6. Reimand J, Isserlin R, Voisin V, Kucera M, Tannus-Lopes C, Rostamianfar A, et al. Pathway enrichment analysis and visualization of omics data using g:Profiler, GSEA, Cytoscape and EnrichmentMap. *Nat Protoc* 2019; **14**: 482–517. https://doi.org/10.1038/s41596-018-0103-9
